# Assessing leaf physiological traits in response to flooding among dominant riparian herbs along the Three Gorges Dam in China

**DOI:** 10.1002/ece3.11533

**Published:** 2024-06-21

**Authors:** Xiaolin Liu, Muhammad Arif, Jie Zheng, Yuanyuan Wu, Yangyi Chen, Jie Gao, Junchen Liu, Li Changxiao

**Affiliations:** ^1^ Key Laboratory of Eco‐Environments in the Three Gorges Reservoir Region (Ministry of Education) College of Life Sciences, Southwest University Chongqing China; ^2^ Biological Science Research Center, Academy for Advanced Interdisciplinary Studies Southwest University Chongqing China

**Keywords:** flooding stress, herb leaf traits, hydro‐fluctuation zone, plant functional types, riparian vegetation

## Abstract

Dams worldwide have significantly altered the composition of riparian forests. However, research on the functional traits of dominant herbs experiencing flooding stress due to dam impoundment remains limited. Given the high plasticity of leaf traits and their susceptibility to environmental influences, this study focuses on riparian herbs along the Three Gorges Hydro‐Fluctuation Zone (TGHFZ). Specifically, it investigates how six leaf physiological traits of leading herbs—carbon, nitrogen, phosphorus, and their stoichiometric ratios—adapt to periodic flooding in the TGHFZ using cluster analysis, one‐way analysis of variance (ANOVA), multiple comparisons, Pearson correlation analysis, and principal component analysis (PCA). We categorized 25 dominant herb species into three plant functional types (PFTs), noting that species from the same family tended to fall into the same PFT. Notably, leaf carbon content (LCC) exhibited no significant differences across various PFTs or altitudes. Within riparian forests, different PFTs employ distinct adaptation strategies: PFT‐I herbs invest in structural components to enhance stress resistance; PFT‐II, mostly comprising gramineous plants, responds to prolonged flooding by rapid growth above the water; and PFT‐III, encompassing nearly all Compositae and annual plants, responds to prolonged flooding with vigorous rhizome growth and seed production. Soil water content (SWC) emerges as the primary environmental factor influencing dominant herb growth in the TGHFZ. By studying the response of leaf physiological traits in dominant plants to artificial flooding, we intend to reveal the survival mechanisms of plants under adverse conditions and lay the foundation for vegetation restoration in the TGHFZ.

## Graphical abstract

Gramineae and Compositae families were significantly represented among the 25 dominant herb species. Annuals and perennials were distributed evenly among herb functional types in riparian forests. Variations in leaf traits among different functional types reveal complex and varied responses to changes in flooding intensity. Plant growth was primarily affected by nitrogen in riparian forests. Soil moisture content dominated environmental factors for herb species growth within the riparian zone.

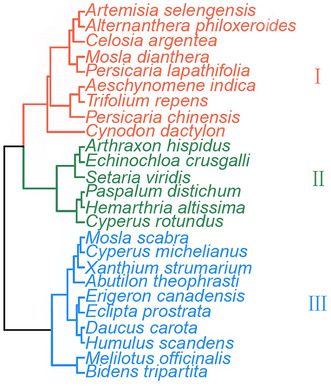

## 1 INTRODUCTION

Multiple aspects of modern life depend on dams as essential components of engineered infrastructure (Arif et al., 2023). The development of these dams has contributed to many conveniences, including improved navigation, seasonal water storage and drainage, and hydroelectric power generation. Despite this, dam construction presents both opportunities and challenges. Dams fundamentally alter rivers, and the effects on their surroundings are long‐lasting (Aguiar et al., 2019). Dams influence river fluctuations by shifting river levels (Bao et al., 2015; Liro, 2019). Therefore, terrestrial and aquatic ecosystems undergo structural and functional changes (Annala et al., 2022; Camarero et al., 2023; Feyissa et al., 2023). Hydrological changes in the drawdown zone affect topography, landforms, hydrology, and sedimentation along riverbanks. This results in soil degradation and a significant decrease in plant diversity within the riparian forest along the drawdown zone. Ultimately, these changes have a profound impact on riparian ecosystem functions and services (Hernandez & Sandquist, 2019). Dam drawdown zones are some of the most degraded ecosystems in the world. The Three Gorges Dam (TGD) exemplifies this phenomenon, creating a water‐fluctuation zone of nearly 350 km^2^ following its completion.

Plant functional types (PFTs) represent groups of plants that share key functional traits, respond to environmental stimuli in a similar manner (Geldenhuys et al., 2022; Hou et al., 2022; Wang, Li, et al., 2023; Wang, Sun, et al., 2023; Zhang et al., 2023), and play analogous roles in significant ecosystem processes (Yan et al., 2019), especially along rivers. Traditionally, PFTs are rooted in plant taxonomy (He et al., 2020), life history, leaf habits (Liu et al., 2023), habitat types, and other such criteria. Although there has been research on plant functional traits within riparian forests along the drawdown zone so far, it has only looked at how functional traits of individual species or a few species change in response to environmental factors (Zhu et al., 2022). While this approach offers valuable insights into how individual species adapt to their surroundings, it lacks the ability to account for uncontrollable factors that drive community‐level responses to the environment. Moreover, individually analyzing multiple species’ functional characteristics within a specific area can be quite laborious. Consequently, grouping multiple species into functional types can simplify, to a certain extent, the understanding of the ecological roles plants play in critical ecosystem processes (Bär Lamas et al., 2016).

The mass ratio hypothesis asserts that ecosystem characteristics are primarily determined by the characteristics of dominant species within a community (Bär Lamas et al., 2016; Zhu et al., 2022). It follows that these dominant species profoundly influence ecosystem dynamics (Cadotte, 2017). The plant functional traits refer to a set of core attributes that are closely related to plant colonization, survival, growth, and senescence (Charles et al., 2022; Zhao et al., 2021). They are frequently employed to elucidate how plants adapt to various environments and respond to external environmental perturbations. Given plants’ immobility, their functional traits are a result of their long‐term evolutionary interactions with their surroundings. Due to species’ varying stress tolerances, they occupy distinct habitats (Wang & Wang, 2023). Furthermore, the environmental filtering effect fosters similarity among plant functional traits residing in the same geographical zone. Consequently, these plants occupy ecological niches that are multiple, overlapping, and staggered within the community (Li, He, et al., 2022). Thus, plant functional traits serve as potent indicators of species distribution factors.

Leaf traits stand as potent proxies for the entire plant and are often indicative of adaptation strategies (Wang et al., 2017). The water level fluctuation zone at an elevation of 145–175 m formed by the special water storage method of the TGD also affects the leaf characteristics of the riparian vegetation in this area. Specifically, plants inhabiting higher elevations contend with less frequent and shorter‐duration floods, while those at lower elevations grapple with more frequent and extended inundations. Low‐ and high‐elevation plants exhibit distinct responses to flood‐related traits, particularly evident in their leaf traits (Li, He, et al., 2022; Li, Zhu, et al., 2022). Plant leaves, as the primary organs for photosynthesis and material production (Tian et al., 2021), are also the organs most directly exposed to the external environment. Consequently, they possess high sensitivity and adaptability to environmental shifts. Leaf nutrient stoichiometry is a vital determinant of plant traits. In the realm of ecology, leaf functional traits serve as an intuitive means to gauge the impact of environmental changes on plant growth and the responses and adaptations of plants to these changes (Sun et al., 2021).

In recent years, as ecological stoichiometry garners increasing attention, leaf stoichiometry, particularly with respect to carbon (C), nitrogen (N), and phosphorus (P), has taken the spotlight in research. These elements serve as the foundational building blocks for a multitude of crucial substances within organisms (Ding et al., 2022) and play pivotal roles in various metabolic activities. For example, C plays a crucial role in forming the structural framework of organisms, while N and P are essential components of biological macromolecules like proteins and nucleic acids, significantly contributing to growth and sustaining normal life functions (Cui et al., 2022). Also, the C:N and C:P ratios in leaves show how well N and P are absorbed during C assimilation (Huang et al., 2019). Leaf N:P ratios are linked to a range of ecological attributes and processes, including plant growth rates and strategies, nutrient availability, ecosystem structure and function, and more (Huang et al., 2019). They can help with restoring riparian forests, managing vegetation, and figuring out how plants will spread in these areas.

Riparian zones are dynamic habitats characterized by spatial and temporal variations, acting as an interface between terrestrial and aquatic ecosystems (Huang et al., 2019). These zones facilitate energy flow, material circulation, and information exchange among species. Notably, hydrological changes influence plant growth and leaf functional characteristics within this habitat (Toner & Keddy, 1997; Vesipa et al., 2016; Zhang et al., 2019). In the particular setting of the Three Gorges Hydro‐Fluctuation Zone (TGHFZ), human‐controlled anti‐seasonal fluctuations in water levels as well as more conventional factors like soil nutrient content, light, and temperature all have an impact on vegetation growth. Plants suffered from serial submergence stress in the TGHFZ, with durations as long as 210 days at depths of up to 30 m (Ding et al., 2021). This unusual water level fluctuation pattern arises from the TGD construction and adheres to the “storing clear water and discharging muddy water” strategy (Chen, Song, et al., 2022; Chen, Wei, et al., 2022; Jiajia et al., 2023). In the backdrop of global climate change and the escalating demands of human society, exemplified by the growing number of dams worldwide, the natural survival of vegetation in its original environment faces significant threats (Oettel et al., 2022; Procknow et al., 2023; Wei et al., 2022). A case in point is the TGHFZ, where most of the original woody plants have vanished due to their inability to tolerate prolonged flooding, leaving behind predominantly annual to perennial herbaceous plants in riparian forests (Zhang & Xie, 2021). Since there has been little research on herb leaf physiological traits at a multispecies level despite long‐term anti‐seasonal flooding, this study explores the relationship between leaf physiological traits and flooding, focusing on functional types of vegetation in TGHFZ because of long‐term anti‐seasonal flooding. The study seeks to address the following scientific inquiries: (1) What are the disparities in herb composition across different functional types? (2) How do the herb leaf physiological traits of each functional type adapt to flooding? (3) What role do environmental factors play in influencing each functional type of dominant herb in riparian forests?

## 2 MATERIALS AND METHODS

### 2.1 Study area

The study sites are situated within the TGHFZ, with coordinates ranging from 28°17′ to 32°05′N latitude and 105°73′ to 111°12′E longitude. This locale is under the influence of a humid subtropical monsoon climate (Teng et al., 2019). The annual average temperature registers at 18.22 ± 0.56°C (mean ± SD), while precipitation ranges from 1000 to 1300 mm annually (Arif et al., 2024). Rainfall is distributed unevenly across the seasons, with 60–80% occurring between April and September (Ye et al., 2020). The TGD area's elevations span from a minimum of 115.2 ± 45.0 m to a maximum of 1926.7 ± 683.8 m. The soils in this area are classified as Entisols (Regosols in Food and Agriculture Organization (FAO) taxonomy) or purple soil and yellow soil in Chinese soil taxonomy (Zheng et al., 2023). The region is characterized by expansive hilly and mountainous terrain, interspersed with smaller flatland areas. This region's land structures exhibit significant vertical variations. Since the completion and activation of the TGD in 2010, the reservoir has been impounded since September each year. It reaches its highest water level at 175 m. By the end of May the following year, the water is gradually released, lowering the lowest water level to 145 m (Su et al., 2020), which formed a water level fluctuation zone with a vertical drop of 30 m and submergence time of 7 months in this area yearly. Prior to the TGD's operation, the reservoir's water level followed a natural rhythm, peaking in summer and hitting its lowest point in winter. However, with the TGD in operation, the water level in the TGD's drawdown area is primarily controlled by human intervention. This results in a higher water level during the winter and a lower one in the summer. This artificial manipulation departs from natural seasonal fluctuations, giving rise to anti‐seasonal water level fluctuations.

### 2.2 Sampling and measurement

**FIGURE 1:**
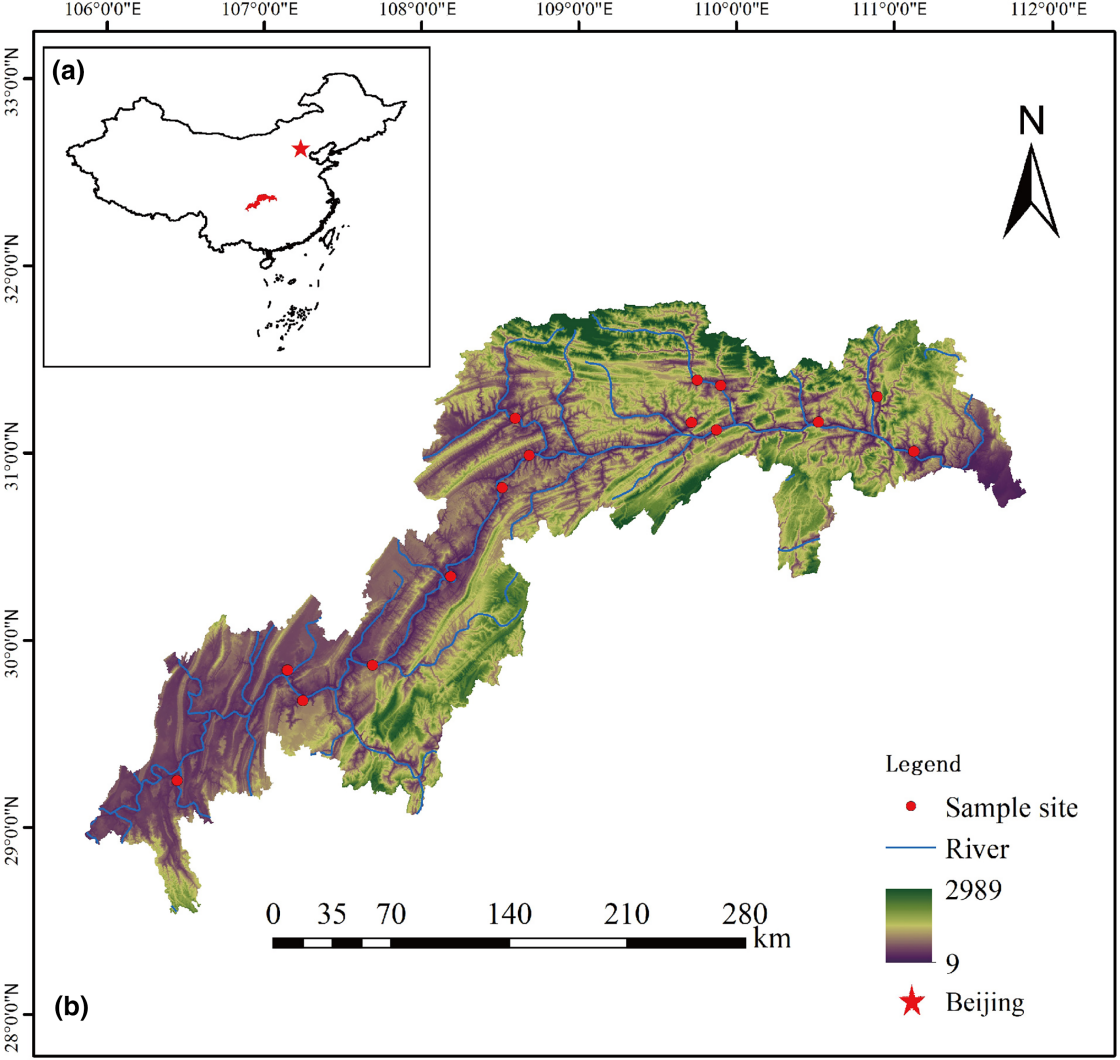
The study site is located in the Three Gorges Reservoir (b) of China (a).

**FIGURE 2:**
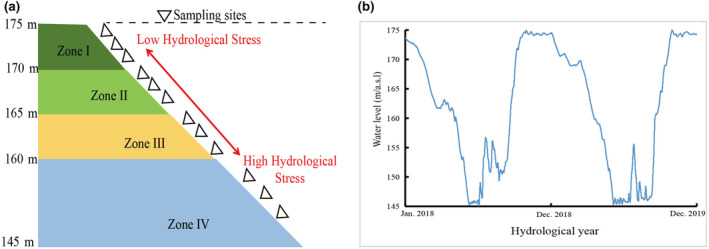
(a) The figure represents the sampling sites across different zones, while (b) pertains to the hydrological conditions in the Three Gorges Hydro‐Fluctuation Zone (TGHFZ) from 2018 to 2019.

Field sampling was conducted during peak plant growth in July and August 2019. In accordance with the vegetation distribution in the TGHFZ, surveys and sampling were undertaken in 15 districts and counties within the region. Of these, Badong, Yiling, and Xingshan are situated in Hubei Province, while the remaining districts and counties are located in Chongqing (see Figure 1). To investigate the impact of varying flood intensities on vegetation and in consideration of the distinctive hydrological rhythm of the study area (refer to Figure 2), we used elevation as a proxy of flooding intensity. The lower the elevation, the longer the period of inundation per year. In addition to the fact that the area with an elevation of 145–165 m is often affected by natural flooding due to the rainy summer climate (Zheng, Arif, Zhang, Yuan, Zhang, Dong, et al., 2021; Zheng, Arif, Zhang, Yuan, Zhang, Li, et al., 2021), the elevation range of 145–175 m was divided into four specific zones: 170 m–175 m (Zone I), 165 m–170 m (Zone II), 160 m–165 m (Zone III), and 145 m–160 m (Zone IV) (Zheng, Arif, Zhang, Yuan, Zhang, Dong, et al., 2021; Zheng, Arif, Zhang, Yuan, Zhang, Li, et al., 2021).

In line with the actual vegetation growth within each surveyed district or county, 50‐m sections were established at different elevations. This design ensured that each elevation range in each district or county was represented, resulting in a total of 100 sections. Within each section, three 2 × 2 m quadrats were randomly selected to assess the vegetation community. Information, such as species name, average height, abundance, coverage, frequency, slope, aspect, elevation, longitude, and latitude of each quadrat, was recorded. Plant coverage was used to determine species dominance, with plants with coverage exceeding 20% in a quadrat considered dominant species. Leaves from these identified dominant species were collected on‐site, labeled, and placed in self‐sealing bags for later laboratory analysis. The collected materials were washed with clean and deionized water, excess surface water was removed using absorbent paper, and the plants were then oven‐dried. After rapid blanching at 105°C, the temperature was maintained at around 80°C to achieve a constant weight. Subsequently, the dried plant samples were crushed and sieved through a 0.25‐mm sieve, sealed, and stored for the determination of plant element content. A total of 257 samples were collected. We used a Vario EL cube CHNOS Element Analyzer from Elementar Analysensysteme GmbH, Germany, to find out how much C and N were present in the leaves. We also used an ICP‐6300 instrument from Thermo Scientific Fisher, USA, to find out how much P was present in the leaves using inductively coupled plasma optical emission spectrometry (ICP‐OES). The C/N, C/P, and N/P values in leaves were calculated. Notably, when the same plant species appeared in multiple transects within the same habitat (i.e., the same elevation area along the same river), it was sampled and tested in only one transect.

To ensure accurate soil property data, the following methodology was employed: Began by clearing away any vegetation and dead leaves from each sample plot. Utilized the “five‐point sampling method” to collect soil samples from 0 to 20 cm depth. This involved extracting soil samples at five evenly spaced points within the sample plot. Once the samples were collected, we thoroughly mixed them to ensure a representative composite sample. Employed the quartering method to obtain a 1 kg subsample from the mixed composite. Simultaneously, measured soil bulk density at the central point of the sample plot using a foil sampler. Placed the soil sample in a marked self‐sealing bag, ensuring proper identification. Transported the soil sample back to the laboratory. In the laboratory, dried the soil sample in an oven at 105°C until a constant weight was achieved. Also, we conducted a range of measurements on the soil sample, including soil total phosphorus (TP), soil available phosphorus (AP), soil total nitrogen (TN), soil ammonium nitrogen (NH_4_^+^–N), soil nitrate nitrogen (NO_3_^−^–N), soil total potassium (TK), soil available potassium (AK), soil water content (SWC), soil bulk density (BD), and soil pH.

### 2.3 Data analysis

To analyze PFT responses to flooding, we employed the following methodology: Species‐level trait data were derived by calculating the mean values of all leaf traits based on the collected replicates. These data included six functional trait values, and 25 species were divided into functional types. To mitigate differences in the range of trait values and ensure that each trait has an equal impact on the cluster analysis results, the leaf trait data for each species were initially logarithmized (log_10_). This standardization step aimed to achieve maximum uniformity among the trait values. R software was used to calculate the distance between various species for cluster analysis. The sum of squares of deviations was employed as a distance metric to determine the relationships and groupings of the dominant species. This was done based on their functional traits. Depending on the distribution and variance homogeneity of the data, one‐way analysis of variance (ANOVA), multiple t‐tests, and the Kruskal–Wallis H test were performed to analyze significant differences in various traits among different PFTs in varying flooded elevation regions. Normality and variance homogeneity tests were conducted prior to the analysis, with data transformation applied when necessary to meet these assumptions. The correlation between leaf traits and environmental variables was assessed using Pearson correlation analysis at 95% confidence level. Principal component analysis (PCA) was employed to explore multivariate covariant relationships among PFTs across functional types. This technique reduced the multidimensional trait space to its first two principal trait axes (Trait first principal component (PC1) and second principal component (PC2)). The primary environmental drivers of multidimensional trait variation were determined by calculating the relative importance of each environmental variable in explaining trait PC1 and PC2. PCA was conducted using the prcomp function, and relative importance was quantified through the “relweights” function. All statistical analyses and data visualizations were carried out using R version 4.2.2 and Excel.

## 3 RESULTS

**FIGURE 3:**
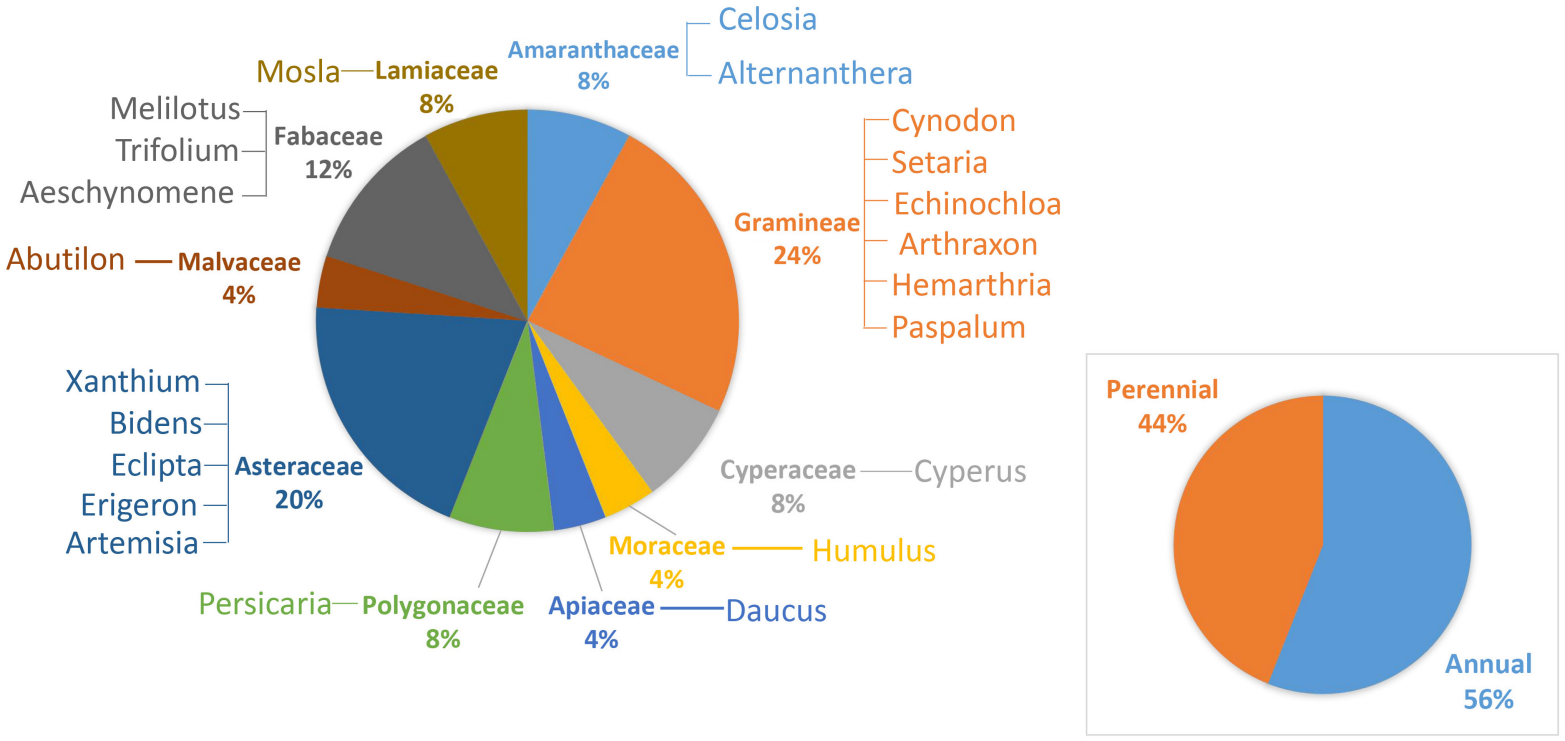
An overview of dominant plant families, genera, and life forms in the study area.

During the field survey in the TGHFZ, a total of 25 dominant herbaceous vegetation species were identified, spanning across 10 different plant families (as detailed in Table 1). Notably, the Gramineae and Compositae families emerged as the dominant ones, representing 24% and 20% of the total, respectively. It is worth highlighting that among this dominant vegetation, 56% consisted of annual plants, signifying the prevalence of these species in the area (as illustrated in Figure 3).

**TABLE 1:** An overview of dominant species selected from the riparian zone of the Three Gorges Hydro‐Fluctuation Zone in China.

Species	Common name	Family	Genus	Life form	Important value	Reference
*Celosia argentea*	Cockscomb	Amaranthaceae	Celosia	Annual	0.1121	Zhu, Chen, Li, and Shao (2020), Ho et al. (2022)
*Alternanthera philoxeroides*	Alligator weed	Amaranthaceae	Alternanthera	Perennial	0.095	Zhu et al. (2022), Nahar et al. (2022)
*Cyperus rotundus*	Nut grass	Cyperaceae	Cyperus	Perennial	0.11	Zhu, Chen, Li, and Shao (2020), Zhu et al. (2022)
*Cyperus michelianus*	Scaly sedge	Cyperaceae	Cyperus	Annual	0.0557	Li, Zhu, et al. (2022)
*Humulus scandens*	Scandent hop	Moraceae	Humulus	Perennial	0.0691	Pan et al. (2019)
*Daucus carota*	Wild carrot	Umbelliferae	Daucus	Perennial	0.0938	Li, Zhu, et al. (2022)
*Polygonum chinensis*	Chinese knotweed	Polygonaceae	Persicaria	Perennial	0.0955	Zhang and Xie (2021)
*Persicaria lapathifolia*	Pale smartweed	Polygonaceae	Persicaria	Annual	0.098	Li, Zhu, et al. (2022)
*Artemisia selengensis*	Mud artemisia	Asteraceae	Artemisia	Perennial	0.08	Dou et al. (2023)
*Erigeron canadensis*	Horseweed	Asteraceae	Erigeron	Annual	0.0621	Zhu, Chen, Li, and Shao (2020), Sun et al. (2021)
*Bidens tripartita*	Burr marigold	Asteraceae	Bidens	Annual	0.1087	Sun et al. (2021)
*Eclipta prostrata*	Eclipta	Asteraceae	Eclipta	Annual	0.0646	Zhu, Chen, Li, and Shao (2020), Li, Zhu, et al. (2022)
*Xanthium strumarium*	Common cocklebur	Asteraceae	Xanthium	Annual	0.165	Chen, Song, et al. (2022), Chen, Wei, et al. (2022), Zhu, Chen, Li, and Shao (2020)
*Abutilon theophrasti*	Piemarker	Malvaceae	Abutilon	Annual	0.1276	Zhu, Chen, Li, and Shao (2020), Li, He, et al. (2022), Li, Zhu, et al. (2022)
*Hemarthria sibirica*	Bullbena	Poaceae	Hemarthria	Perennial	0.137	Dou et al. (2023)
*Arthraxon hispidus*	Hairy jointgrass	Poaceae	Arthraxon	Annual	0.1593	Sun et al. (2021)
*Paspalum distichum*	Dallisgrass	Poaceae	Paspalum	Perennial	0.1307	Zhang and Xie (2021)
*Setaria viridis*	Green bristlegrass	Poaceae	Setaria	Annual	0.1011	Chen, Song, et al. (2022), Chen, Wei, et al. (2022), Zhu, Chen, Li, and Shao (2020)
*Cynodon dactylon*	Bermudagrass	Poaceae	Cynodon	Perennial	0.3259	Zhu, Chen, Zhang, et al. (2020)
*Echinochloa crusgalli*	Barnyardgrass	Poaceae	Echinochloa	Annual	0.1052	Chen, Song, et al. (2022), Chen, Wei, et al. (2022), Zhu, Chen, Li, and Shao (2020)
*Aeschynomene indica*	Indian jointvetch	Fabaceae	Aeschynomene	Annual	0.07466	Xuan et al. (2004)
*Melilotus officinalis*	Yellow sweet clover	Fabaceae	Melilotus	Perennial	0.1097	Sun et al. (2021)
*Trifolium repens*	White clover	Fabaceae	Trifolium	Perennial	0.1368	Zhang and Xie (2021)
*Mosla dianthera*	Miniature beefsteakplant	Labiatae	Mosla	Annual	0.1315	Kim et al. (2000)
*Mosla scabra*	Herba Moslae	Labiatae	Mosla	Annual	0.0606	Bhatt et al. (2022), Li et al. (2010)

### 3.1 Vegetation composition of various functional types

**FIGURE 4:**
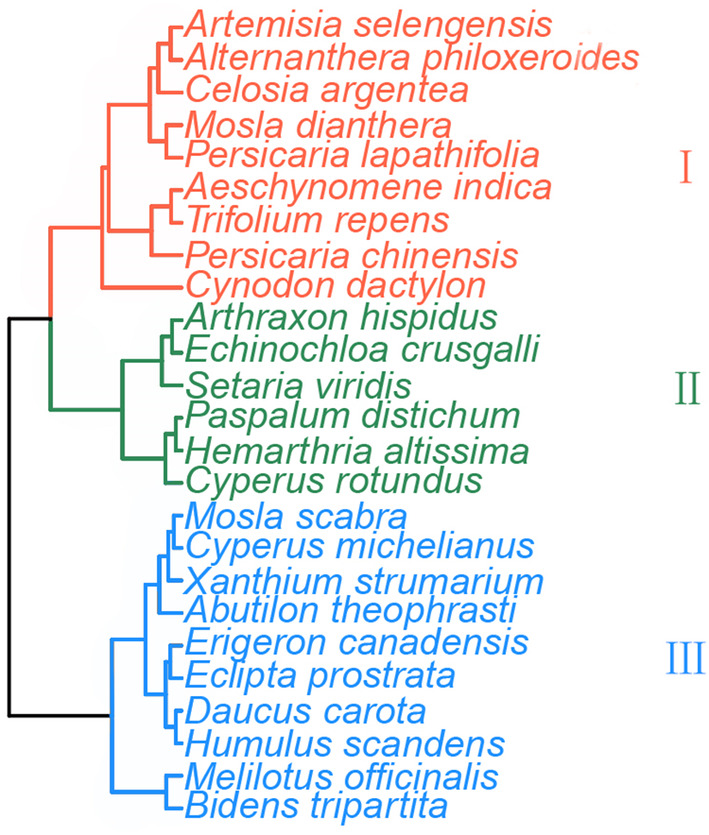
Cluster diagram of 25 dominant plants in the TGHFZ. Light pink: Function Type I, Green: Function Type II, and Blue: Function Type III.

Cluster analysis divided the 25 most common herb plants into three clear functional groups based on measurements of six leaf functional traits (leaf carbon, nitrogen, phosphorus contents and their ratios to each other) (Figure 4). Notably, the vast majority of Compositae and Gramineae species were predominantly found in functional types III and II, respectively. These species accounted for 83.33% and 80% of the total number of PFT III and II, respectively. It is worth highlighting that the dominant species in the riparian zone, *Cynodon dactylon*, was clustered and separated from the main Gramineae group. Most functional type III species grouped by cluster analysis are annual plants, except *Humulus scandens* and *Osmanthus officinalis*. In contrast, the proportion of annual and perennial plants in the other two functional types was more evenly distributed. Functional types I and II, for example, contained 44.4% and 50% annual plants, respectively (Figure 4).

### 3.2 Response of each functional group to flooding

**FIGURE 5:**
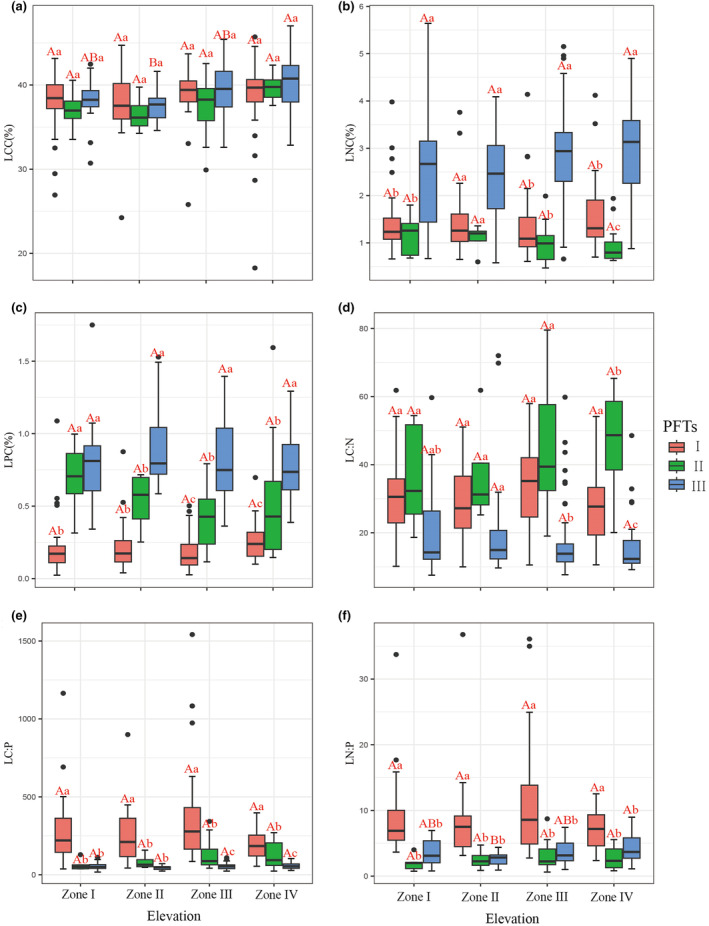
Response of different functional types to flooding. Different capital letters indicate significant differences among functional types at varying altitudes, while different lowercase letters represent significant differences among functional types at the same altitude. LCC, Leaf Carbon Content; LNC, Leaf Nitrogen Content; LPC, Leaf Phosphorus Content; LC:N, Leaf Carbon‐to‐Nitrogen Ratio; LC:P, Leaf Carbon‐to‐Phosphorus Ratio; LN:P, Leaf Nitrogen‐to‐Phosphorus Ratio.

The analysis of leaf carbon content (LCC) across different elevations reveals that there were no significant differences in LCC among the various functional types. Specifically, there were no significant differences in LCC between functional types I and II at different elevations. However, LCC for functional type III was significantly lower at elevation II than at several other elevations (Figure 5a, *p* < .05). Leaf nitrogen content (LNC) was not significant at different elevations for each functional type. Even so, there were big differences in LNC between functional types at all other elevations. For example, LNC for functional type III was much higher than those for the other two types (Figure 5b, *p* < .05). Leaf phosphorus content (LPC), leaf carbon–nitrogen ratio (LC:N), and leaf carbon–phosphorus ratio (LC:P) did not exhibit significant differences at different elevations within each functional type (Figure 5c–e, *p* > .05). However, it is pertinent to note that the leaf nitrogen–phosphorus ratio (LN:P) for functional type III was significantly lower at elevation II than other elevations (Figure 5f, *p* < .05).

The trends in LNC and LPC for functional type I showed a slight increase with rising flooding intensity, as depicted in Figure 5b (ranging from 1.472% to 1.581%) and Figure 5c (ranging from 0.23% to 0.252%), respectively. In contrast, LPC and LC:N for functional type II initially decreased and then increased with increasing flooding intensity (Figure 5c,d). However, LNC exhibited the opposite pattern, decreasing as flooding intensity rises (Figure 5b). The LC:P and LN:P exhibited consistent changes with flooding intensity (Figure 5e,f). Functional type III showed a distinct pattern. LCC, LNC, and LN:P exhibited a trend of decreasing first and then increasing with increasing flooding intensity (Figure 5a,b,f), while LPC displayed a reverse trend, decreasing initially and then increasing (Figure 5c). LC:N, however, decreased as the flooding intensity increases. These variations in leaf traits reflected the complex responses of different functional types to flooding intensity changes.

### 3.3 Effects of environmental factors on various functional types

**FIGURE 6:**
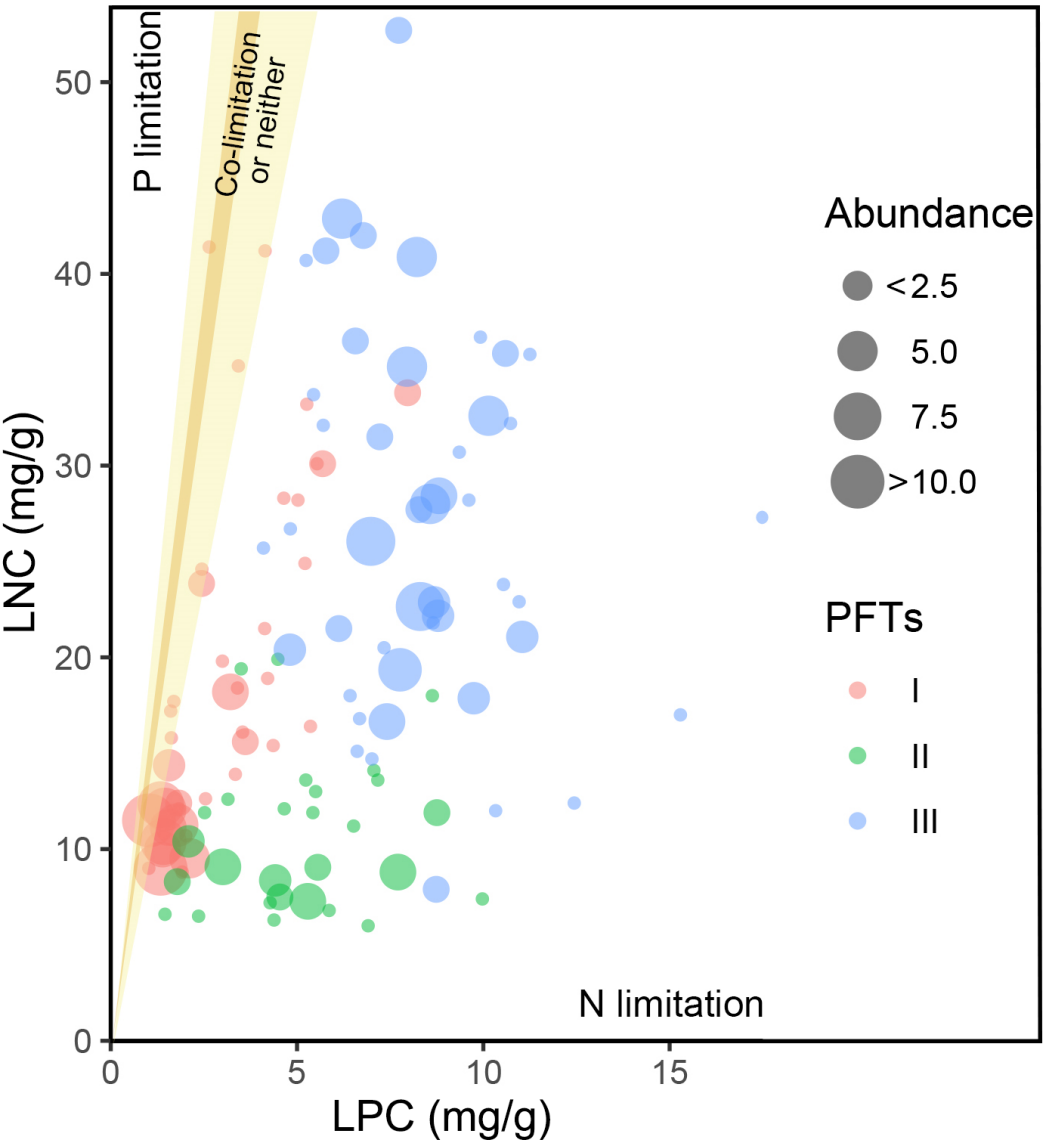
Leaf N:P ratios of 25 TGHFZ species. Circle size indicates species abundance. Yellow shading represents two commonly used leaf N:P thresholds for assessing nutrient limitations: 14:1 and 16:1 (Cui et al., 2022).

The LN:P ratio is commonly considered a straightforward indicator of plant nutrient limitations. In the TGHFZ, the average LN:P for the 25 herb species was 3.56 ± 0.26. This ratio exhibited a considerable range, from 0.742 in *Echinochloa crusgalli* to 15.67 in *Aeschynomene indica* L. Species within functional types II and III were primarily constrained by N alone, which set them apart from functional type I. A small fraction of species within functional type I (constituting 14.63% of functional type I species) were either limited by both N and P elements or influenced by other factors (Figure 6). This highlighted the varied nutrient constraints and ecological niches of different plant species within riparian forests.

**FIGURE 7:**
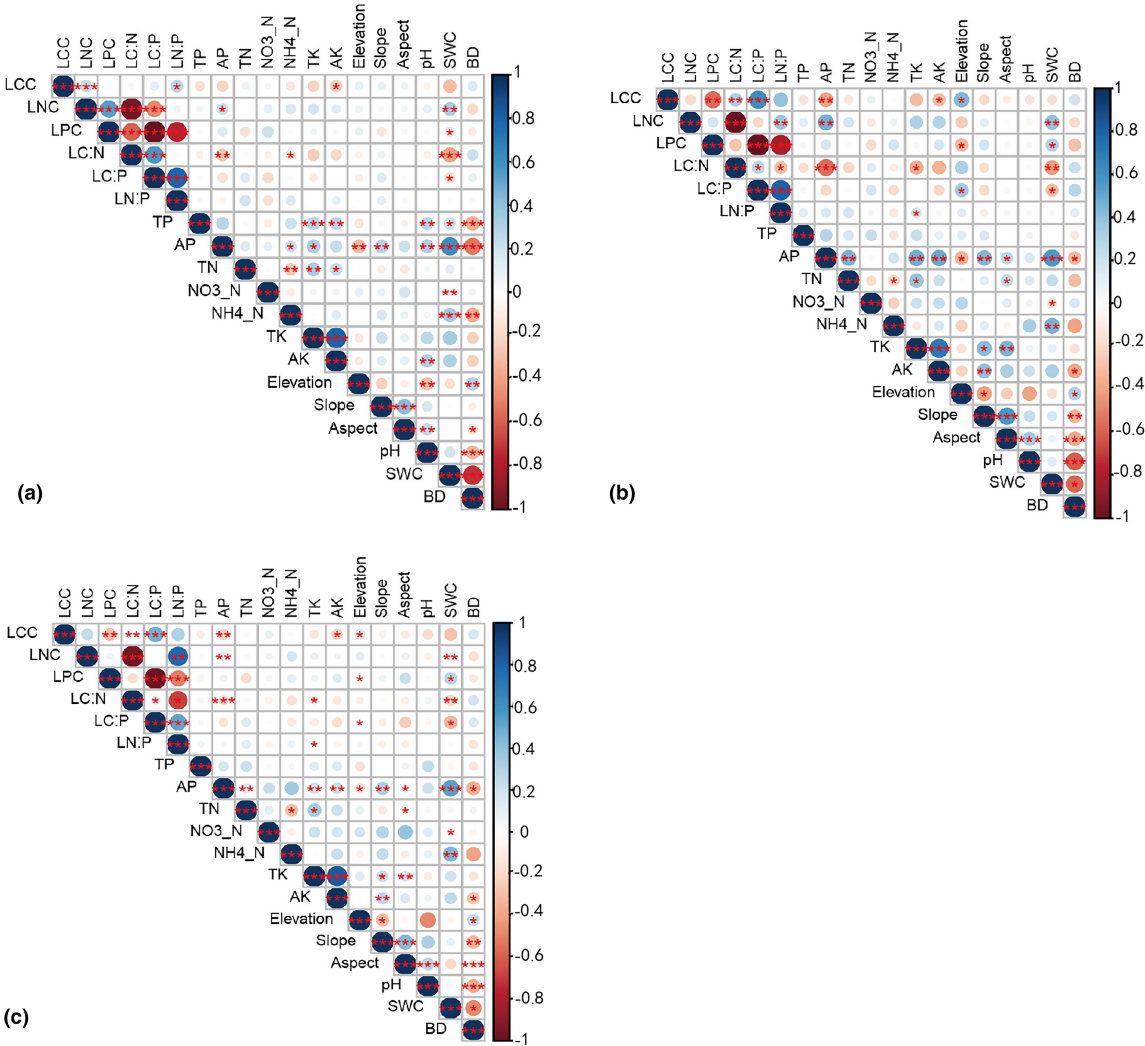
Correlation between leaf traits of each functional type and environmental factors. a = Functional Type I, b = Functional Type II, and c = Functional Type III. Leaf traits include LCC, LNC, LPC, LC:N, LC:P, and LN:P, and environmental factors encompass TP (soil total phosphorus), AP (available phosphorus), TN (total nitrogen), NO_3_^−^–N (nitrate nitrogen), NH_4_^+^–N (ammonium nitrogen), TK (soil total potassium), AK (soil available potassium), SWC (soil water content), and BD (soil bulk density).

There were strong correlations (*p* < .001) between the six leaf traits and the environmental factors linked to them. The leaf traits of different types of plants that perform different things were also strongly correlated (Figure 7). SWC exhibited varying degrees of correlation with leaf physiological traits. In functional type I, AP had a strong negative relationship with LC:N (*p* < .01), while LC:P and LN:P had highly positive relationships with ammonium nitrogen (*p* < .05 and *p* < .01, respectively) (Figure 7a). With increasing SWC, LCC and LC:N decreased, while LNC increased. TK had a significant negative impact on LCC and LC:N. Both AP and TK had substantial adverse effects on LCC and LC:N for functional type II, but they had profound positive effects on LNC (Figure 7b). Notably, AP had a more pronounced effect than TK. This consistency in the effects of AP and TK on leaf physiological traits could be attributed to the highly significant positive correlation between AP and TK (*p* < .001). It is worth noting that there was no correlation between soil N forms and the six leaf physiological traits under study. In functional type III, there was a substantial negative correlation between nitrate nitrogen and LC:N, as well as a significant negative correlation between AK and LCC (Figure 7c). These findings illustrated the intricate relationships between environmental factors and leaf traits in different functional plant types. They highlighted the distinct responses of each type to varying ecological conditions.

**FIGURE 8:**
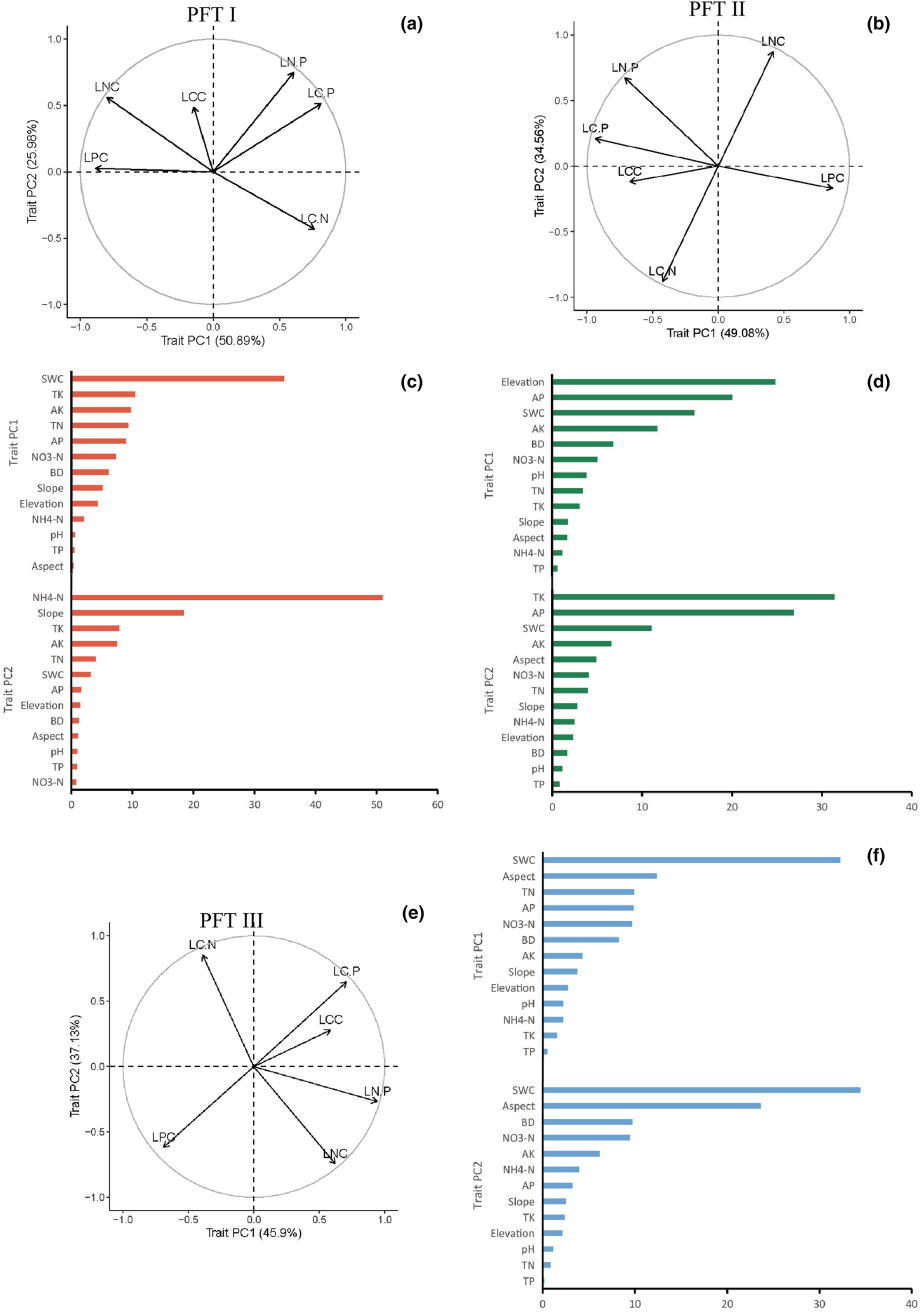
Principal component analysis (PCA) among functional types and correlation strength between the first two principal component axes and environmental factors (a, b, e). The first two principal trait axes (traits PC1 and PC2) for Functional Types I, II, and III, respectively. Solid arrows indicate the direction and weighting of vectors representing the six plant traits (c, d, f). Relative contributions of environmental variables driving spatial patterns of the first two principal trait axes for Functional Types I, II, and III, respectively.

Principla component analysis (PCA) identified two primary trait axes that explained the complex variations in multidimensional traits among different PFTs (Figure 8). These two principal trait axes collectively accounted for 76.87% (PC1 = 50.89%, PC2 = 25.98%) of the total multidimensional trait variation among PFTs (Figure 8a), 83.64% (PC1 = 49.08%, PC2 = 34.56%) (Figure 8b), and 83.03% (PC1 = 45.9%, PC2 = 37.13%) (Figure 8e). In PFT I, the trait PC1 was primarily associated with variations in LC:P and LC:N (Figure 8a). These traits were significantly influenced by SWC and soil TK (Figure 8c), with SWC contributing 35% to PC1 determination. Trait PC2 in PFT I aligned with LN:P and LNC (Figure 8a), with NH_4_–N and slope playing significant roles in their variation (Figure 8c). NH_4_–N was the most prominent factor contributing to PC2, accounting for 51% of its determination. In PFT II, trait PC1 aligned most strongly with LPC and LC:P, while trait PC2 aligned with LNC and LC:N (Figure 8b). Elevation and soil AP were the key factors influencing these traits. Elevation accounted for 25% of PC1's determination, while soil AP contributed 20%. For PC2, soil TK and soil AP played significant roles, with TK contributing 31% and soil AP contributing 27% (Figure 8d). In PFT III, trait PC1 was primarily associated with variations in LN:P and LC:P, while trait PC2 aligned with LC:N and LNC (Figure 8e). SWC and aspect were the most influential factors for these traits, with SWC contributing 32% to PC1 and 34% to PC2. Aspect also played a significant role, contributing 12% to PC1 and 24% to PC2 (Figure 8f). These findings highlighted the key environmental factors driving variations in leaf traits among different functional types, providing insight into the complex interplay between plant characteristics and ecological conditions.

## 4 DISCUSSION

### 4.1 Differences in vegetation composition of various functional types

Cluster analysis was employed to categorize the functional types of 25 herb species gathered from the field. This resulted in the identification of three distinct PFTs. Notably, the two predominant families (Asteraceae and Poaceae) were grouped into the same functional type. This suggests that within these families, plants tend to exhibit similar traits and characteristics when adapting to long‐term, periodic flooding environments caused by artificial storage adjustment. However, plants of the same family can differentiate in response to environmental changes. For example, *Cynodon dactylon*, a dominant member of the Gramineae family, was classified outside its family due to fluctuating water levels in the TGD. This can be attributed to the unique peroxide scavenging mechanism of *Cynodon dactylon* (Zhu, Chen, Li, & Shao, 2020), being able to live in the flooded environment caused by the long‐term water impoundment of the TGD from the end of September to the end of May the following year (Ding et al., 2021). It also has strong homeostasis, low nutrient needs, and high nutrient utilization efficiency (Li et al., 2021). These factors help it do well as a rare “amphibious” plant in the TGHFZ, which is spread out at different elevations.

Considering the predominantly single‐family and single‐genus distribution of vegetation (Rao et al., 2023; Guo et al., 2018), many plants from the same family were categorized into the same functional type. It is evident that the environment acts as a filter (Zheng, Arif, Zhang, Yuan, Zhang, Dong, et al., 2021; Zheng, Arif, Zhang, Yuan, Zhang, Li, et al., 2021), screening species based on their similarity in traits. This contributes to the diversity of species in the TGD area. In essence, species have evolved similar survival strategies to adapt to artificial flooded environments. However, the relationship between the three functional types, as determined by the analysis of six leaf traits in this study, and the life form of the plants remains somewhat unclear. Therefore, further research is needed to elucidate this relationship.

### 4.2 Adaptation of different functional types to artificial flooding

Plant leaves' functional traits serve as a crucial link between plants and their environment (Sun et al., 2017). These traits reflect plants' diverse physiological and ecological functions. LCC is a key indicator of leaf construction and defense capabilities (Wang & Qin, 2020; Xia et al., 2020). In our study area, we observed no significant differences in LCC among different functional types at varying altitudes (*p* > .05). Surprisingly, LCC showed the least variation among the six leaf functional traits, which shows that the hydrological fluctuation zone formed by the artificial water diversion of the TGD has a weak impact on LCC, and the LCC of vegetation in the TGHFZ has strong internal homeostasis. It is in line with the findings of Huang et al. (2019) research. According to the relative growth rate hypothesis (Rees & Raven, 2021), plants with vigorous growth, particularly those with fast growth rates, tend to have higher N and P content. This is due to the involvement of N and P in the synthesis of protein nucleic acids. Fast‐growing plants have a substantial need for ribosomal RNA (rRNA) synthesis, resulting in higher P content than slower‐growing plants (Hu et al., 2020; Tian et al., 2021).

Functional type III is characterized by a relatively high proportion of annual plants. The water diversion pattern of the TGD's winter storage and summer discharge is consistent with the growth period of most vegetation (Ding et al., 2021), and the brief exposure of the hydrological fluctuation zone provides a habitat for the growth of annual plants. Annual vegetation completes its life cycle during this period in order to respond to prolonged flooding by forming seeds (Zhu, Chen, Zhang, et al., 2020). Influenced by their high N and P content, the leaf C‐to‐N ratio (LC:N) and leaf C‐to‐P ratio (LC:P) of functional type III were significantly lower than those of the other two functional types. LC:N and LC:P metrics indicate a plant's ability to assimilate C when absorbing nutrients, reflecting plant nutrient utilization efficiency (Dou et al., 2023). Additionally, they represent the level of C sequestration capacity within plant communities. LC:N can also be used to characterize plant growth rates (Yan et al., 2019). Plants in the fluctuation zone are affected by long‐term artificial flooding, and the plant's access to resources such as light and oxygen has become the main limiting factor restricting the growth of this area (Fischer et al., 2016; Purcell et al., 2019). Therefore, plants in the study area accelerate their growth to obtain limited resources, such as light, to avoid harm to themselves caused by unfavorable environments (Lan et al., 2019). In functional type II, which primarily consists of gramineous plants (except Bermudagrass), a higher LC:N suggests a faster growth rate. This implies that the gramineous plants in the study area have adopted an “escape” strategy. They respond to flooding stress by rapidly growing out of the water, a strategy in line with Huang et al. (2019).

Functional type III is mostly made up of Asteraceae plants, which are suitable because some can reproduce through rhizomes in wet places (Zhang et al., 2022; Zhu, Chen, Zhang, et al., 2020). This ability allows them to thrive in locations with ample soil nutrients, making them less susceptible to long‐term flooding caused by the impoundment of the TGD. Within the TGHFZ, areas with lower elevations are subjected to longer flooding times and deeper flooding depths (Zheng, Arif, Zhang, Yuan, Zhang, Dong, et al., 2021; Zheng, Arif, Zhang, Yuan, Zhang, Li, et al., 2021). In zone II, while functional type III plants endure less flooding stress than zone III, the vegetation in this area is more abundant, leading to heightened biological competition. This competition results in the lowest LCC and LNC. LNC and LPC also provide insights into plants’ photosynthetic capacity. At lower altitudes, where water obstructs light penetration, photosynthesis is significantly limited due to reduced light availability. Nevertheless, despite these constraints, functional types I and II increase their investment in C content within structural components to withstand external flooding stress. This results in consistent LCC across different elevation zones. As a result, functional types I and III employ defensive strategies.

### 4.3 The impact of environmental factors on the dominant vegetation

As per two widely recognized leaf N:P threshold ratio hypotheses (Kong et al., 2020; Li et al., 2017; 2019), when the leaf N:P ratio falls below 14 (or 10), plant growth is primarily limited by N. Conversely, when the leaf N:P ratio exceeds 16 (or 20), plant growth is limited by P. When the ratio falls between these values, growth is constrained by both elements. In the TGHFZ, most species are limited to N elements (Figure 6). These findings are consistent with those of Lu's research in 2019 (Lu, 2019).

The correlation between soil N and P content and the leaf physiological traits of various functional plants is weak. This is different from most of the previous studies that concluded that C, N, and P in plants are mainly derived from the soil, that leaf C, N, and P contents are correlated with soil C, N, and P contents, and that the level of soil N and P contents directly affects leaf N and P contents (Yuan et al., 2022), indicating a shifting coupling relationship between soil and plant nutrients in extreme habitats. Under the special water storage mode of the TGD, prolonged flooding in the hydro‐fluctuation zone shows a strong correlation with the leaf physiological traits of dominant vegetation in the riparian forests (Figures 7 and 8), suggesting that leaf traits in the study area are driven by SWC. This finding aligns with research conducted by Li, He, et al. (2022), Li, Zhu, et al. (2022), Rao et al. (2023) and You et al. (2022). Leaf traits in functional type II are predominantly driven by elevation, whereas functional types I and III are influenced by SWC. This underscores the fact that leaf traits in different functional types are shaped by distinct environmental factors (Figure 8), indicating varying adaptation strategies to the long‐term flooded environment formed under the artificially controlled impoundment of the TGD. Each leaf trait is interdependent and functions collectively to sustain normal growth and development throughout the plant's life cycle.

## 5 CONCLUSION

The clustering and categorization of dominant herbaceous plants in the TGHFZ revealed interesting patterns. Plants within the same family are often grouped into the same functional type, underscoring the influence of shared genetic traits. However, the adaptation strategies of individual species to the changing environment can lead to instances where plants from the same family diverge and fall outside the typical functional type of their family. Furthermore, environmental filtering may result in different families and species being classified within the same functional type. Functional type I plants enhance their resilience to long‐term flooding by increasing structural substance production. In contrast, functional type II plant employs an “escape” strategy to cope with prolonged flooding. Functional type III plant completes its life cycle within a short exposure period, producing seeds that can withstand extended flooding. The environment plays a crucial role in shaping plant traits. This leads to similar traits among various herbaceous plants in the TGHFZ as they adapt to periodic flooding. In the context of prolonged extreme flooding, the relationship between leaf physiological traits and soil nutrients changes. Soil water content emerges as a significant driver of leaf physiological traits in TGHFZ vegetation. Therefore, plant growth is jointly regulated by the dominant plants in the water‐fluctuation zone of the TGD by balancing the physiological characteristics of their leaves.

## AUTHOR CONTRIBUTIONS

**Xiaolin Liu:** Conceptualization (equal); data curation (equal); formal analysis (equal); investigation (equal); methodology (equal); software (equal); validation (equal); visualization (equal); writing – original draft (equal). **Muhammad Arif:** Conceptualization (equal); data curation (equal); formal analysis (equal); funding acquisition (equal); investigation (equal); methodology (equal); project administration (equal); resources (equal); software (equal); validation (equal); visualization (equal); writing – original draft (equal); writing – review and editing (equal). **Jie Zheng:** Data curation (equal); investigation (equal); methodology (equal); visualization (equal). **Yuanyuan Wu:** Data curation (equal); investigation (equal); methodology (equal); visualization (equal). **Yangyi Chen:** Data curation (equal); investigation (equal); methodology (equal); visualization (equal). **Jie Gao:** Data curation (equal); investigation (equal); methodology (equal); visualization (equal). **Junchen Liu:** Data curation (equal); investigation (equal); methodology (equal); visualization (equal). **Li Changxiao:** Funding acquisition (equal); investigation (equal); methodology (equal); project administration (equal); resources (equal); supervision (equal); writing – review and editing (equal).

## FUNDING INFORMATION

This work was supported by the Foreign Young Talent Program (Program number: QN2022168001L); Chongqing Housing and Urban–rural Construction Committee (Program number: Chengkezi‐2022‐6‐3); Chongqing Municipality Key Forestry Research Project (2021‐9); and Forestry Extension Project of China Central Finance (No. Yulinketui 2023‐8).

## CONFLICT OF INTEREST STATEMENT

The authors declare that there is no conflict of interest that could be perceived as prejudicing the impartiality of the research reported.

## Supporting information


Data S1.

Data S2.

## Data Availability

The data and codes that support the findings of this study are provided as Data S1 and S2.

## References

[ece311533-bib-0001] Aguiar, F. C. , Fernandes, M. R. , Martins, M. J. , & Ferreira, M. T. (2019). Effects of a large irrigation reservoir on aquatic and riparian plants: A history of survival and loss. Watermark, 11, 2379. 10.3390/w11112379

[ece311533-bib-0002] Annala, M. J. , Lehosmaa, K. , Ahonen, S. H. K. , Karttunen, K. , Markkola, A. M. , Puumala, I. , & Mykrä, H. (2022). Effect of riparian soil moisture on bacterial, fungal and plant communities and microbial decomposition rates in boreal stream‐side forests. Forest Ecology and Management, 519, 120344. 10.1016/j.foreco.2022.120344

[ece311533-bib-0003] Arif, M. , Jie, Z. , Behzad, H. M. , & Changxiao, L. (2023). Assessing the impacts of ecotourism activities on riparian health indicators along the three gorges reservoir in China. Ecological Indicators, 155, 111065. 10.1016/j.ecolind.2023.111065

[ece311533-bib-0004] Arif, M. , Petrosillo, I. , & Changxiao, L. (2024). Effects of changing riparian topography on the decline of ecological indicators along the drawdown zones of long rivers in China. Frontiers in Forests and Global Change, 7, 1293330. 10.3389/ffgc.2024.1293330

[ece311533-bib-0005] Bao, Y. , Gao, P. , & He, X. (2015). The water‐level fluctuation zone of three gorges reservoir—A unique geomorphological unit. Earth‐Science Reviews, 150, 14–24. 10.1016/j.earscirev.2015.07.005

[ece311533-bib-0006] Bär Lamas, M. I. , Carrera, A. L. , & Bertiller, M. B. (2016). Meaningful traits for grouping plant species across arid ecosystems. Journal of Plant Research, 129, 449–461. 10.1007/s10265-016-0803-6 26897637

[ece311533-bib-0007] Bhatt, A. , Caron, M. M. , Chen, X. , Yu, D. , & Niu, Y. (2022). Effect of temperature, light and storage on seed germination of *Salvia plebeia* r.Br., *Leonurus japonicus Houtt*., *Mosla scabra* (Thunb.) C.Y.Wu & H.W.Li and *Perilla frutescens* (L.) Britton. Journal of Applied Research on Medicinal and Aromatic Plants, 31, 100402. 10.1016/j.jarmap.2022.100402

[ece311533-bib-0008] Cadotte, M. W. (2017). Functional traits explain ecosystem function through opposing mechanisms. Ecology Letters, 20, 989–996. 10.1111/ele.12796 28639274

[ece311533-bib-0009] Camarero, J. J. , Colangelo, M. , & Rodriguez‐Gonzalez, P. M. (2023). Tree growth, wood anatomy and carbon and oxygen isotopes responses to drought in Mediterranean riparian forests. Forest Ecology and Management, 529, 120710. 10.1016/j.foreco.2022.120710

[ece311533-bib-0010] Charles, B. , Chase, M. H. , Pociask, G. , Bhattarai, R. , & Matthews, J. W. (2022). Can functional leaf traits be used for monitoring wetland restoration? A comparison between commonly used monitoring metrics and functional leaf traits. Ecological Indicators, 140, 109032. 10.1016/j.ecolind.2022.109032

[ece311533-bib-0011] Chen, Y.‐H. , Wei, G.‐W. , Cui, Y. , & Luo, F.‐L. (2022). Nutrient inputs alleviate negative effects of early and subsequent flooding on growth of *Polygonum hydropiper* with the aid of adventitious roots. Frontiers in Plant Science, 13, 1–11. 10.3389/fpls.2022.919409 PMC9355131 35937344

[ece311533-bib-0012] Chen, Z. , Song, H. , Arif, M. , & Li, C. (2022). Effects of hydrological regime on *Taxodium ascendens* plant decomposition and nutrient dynamics in the three gorges reservoir riparian zone. Frontiers in Environmental Science, 10, 990485. 10.3389/fenvs.2022.990485 PMC8187854 34122483

[ece311533-bib-0013] Cui, E. , Lu, R. , Xu, X. , Sun, H. , Qiao, Y. , Ping, J. , Qiu, S. , Lin, Y. , Bao, J. , Yong, Y. , Zheng, Z. , Yan, E. , & Xia, J. (2022). Soil phosphorus drives plant trait variations in a mature subtropical forest. Global Change Biology, 28, 3310–3320. 10.1111/gcb.16148 35234326

[ece311533-bib-0014] Ding, D. , Arif, M. , Liu, M. , Li, J. , Hu, X. , Geng, Q. , Yin, F. , & Li, C. (2022). Plant‐soil interactions and C:N:P stoichiometric homeostasis of plant organs in riparian plantation. Frontiers in Plant Science, 13, 1–17. 10.3389/fpls.2022.979023 PMC9376457 35979078

[ece311533-bib-0015] Ding, D. , Liu, M. , Arif, M. , Yuan, Z. , Li, J. , Hu, X. , Zheng, J. , & Li, C. (2021). Responses of ecological stoichiometric characteristics of carbon, nitrogen, and phosphorus to periodic submergence in mega‐reservoir: Growth of *Taxodium distichum* and *Taxodium ascendens* . Plants, 10(10), 2040. 34685849 10.3390/plants10102040 PMC8540895

[ece311533-bib-0016] Dou, W. , Jia, W. , Zhang, J. , Yi, X. , Wen, Z. , Wu, S. , & Ma, M. (2023). Research progress of vegetation status, adaptive strategies and ecological restoration in the water‐level fluctuation zone of the Three Gorges Reservoir. Chinese Journal of Ecology, 42, 208–218. 10.13292/j.1000-4890.202301.018

[ece311533-bib-0017] Feyissa, A. , Chen, R. , & Cheng, X. (2023). Afforestation inhibited soil microbial activities along the riparian zone of the upper Yangtze River of China. Forest Ecology and Management, 538, 120998. 10.1016/j.foreco.2023.120998

[ece311533-bib-0018] Fischer, F. M. , Wright, A. J. , Eisenhauer, N. , Ebeling, A. , Roscher, C. , Wagg, C. , Weigelt, A. , Weisser, W. W. , & Pillar, V. D. (2016). Plant species richness and functional traits affect community stability after a flood event. Philosophical Transactions of the Royal Society, B: Biological Sciences, 371(1694), 20150276. 10.1098/rstb.2015.0276 PMC4843697 27114578

[ece311533-bib-0019] Geldenhuys, M. , Gaigher, R. , Pryke, J. S. , & Samways, M. J. (2022). Vineyards compared to natural vegetation maintain high arthropod species turnover but alter trait diversity and composition of assemblages. Agriculture, Ecosystems & Environment, 336, 108043. 10.1016/j.agee.2022.108043

[ece311533-bib-0020] Guo, Y. , Yang, S. , Shen, Y. , Xiao, W. , & Cheng, R. (2018). Composition and niche of the existing herbaceous plants in the water‐level‐fluctuating zone of the three gorges reservoir area, China. Journal of Applied Ecology, 29, 3559–3568. 10.13287/j.1001-9332.201811.006 30460802

[ece311533-bib-0021] He, M. , Yan, Z. , Cui, X. , Gong, Y. , Li, K. , & Han, W. (2020). Scaling the leaf nutrient resorption efficiency: Nitrogen vs phosphorus in global plants. Science of the Total Environment, 729, 138920. 10.1016/j.scitotenv.2020.138920 32371208

[ece311533-bib-0022] Hernandez, R. R. , & Sandquist, D. R. (2019). A dam in the drylands: Soil geomorphic treatments facilitate recruitment of the endangered Santa Ana River woolly star. Ecosphere, 10, e02621. 10.1002/ecs2.262

[ece311533-bib-0023] Ho, J.‐T. , Liang, C.‐C. , & Chen, P. J. (2022). First report of root‐knot nematode meloidogyne enterolobii on cockscomb (*Celosia argentea* var. *cristata*) in Taiwan. Plant Disease, 106, 2126. 10.1094/pdis-10-21-2126-pdn

[ece311533-bib-0024] Hou, L. , Kong, W. , Qiu, Q. , Yao, Y. , Bao, K. , Zhang, L. , Jia, H. , Vasenev, I. , & Wei, X. (2022). Dynamics of soil N cycling and its response to vegetation presence in an eroding watershed of the Chinese loess plateau. Agriculture, Ecosystems & Environment, 336, 108020. 10.1016/j.agee.2022.108020

[ece311533-bib-0025] Hu, C. , Li, F. , Yang, N. , Xie, Y.‐H. , Chen, X.‐S. , & Deng, Z.‐M. (2020). Testing the growth rate hypothesis in two wetland macrophytes under different water level and sediment type conditions. Frontiers in Plant Science, 11, 1–12. 10.3389/fpls.2020.01191 32849739 PMC7419612

[ece311533-bib-0026] Huang, D. , Wang, D. , & Ren, Y. (2019). Using leaf nutrient stoichiometry as an indicator of flood tolerance and eutrophication in the riparian zone of the Lijang River. Ecological Indicators, 98, 821–829. 10.1016/j.ecolind.2018.11.064

[ece311533-bib-0027] Jiajia, L. , Arif, M. , & Changxiao, L. (2023). Rare methanotrophs adapt to broader environmental gradients than abundant methanotrophs in the riparian zone of the three gorges reservoir. Land Degradation and Development, 35, 249–263. 10.1002/ldr.4913

[ece311533-bib-0028] Kim, T. H. , Thuy, N. T. , Shin, J. H. , Baek, H. H. , & Lee, H. J. (2000). Aroma‐active compounds of miniature Beefsteakplant (*Mosla dianthera maxim*.). Journal of Agricultural and Food Chemistry, 48(7), 2877–2881. 10.1021/jf000219x 10898640

[ece311533-bib-0029] Kong, W. , Yuan, X. , Lu, H. , Chen, X. , Liu, T. , Gong, X. , & Wang, X. (2020). Ecological stoichiomentry of the representative herbaceous plants in the littoral zone of Peng Xi River in three gorges Reservior. Journal of Chongqing Normal University (Natural Science), 37, 73–82.

[ece311533-bib-0030] Lan, Z. , Huang, H. , Chen, Y. , Liu, J. , Chen, J. , Li, L. , Li, L. , Jin, B. , & Chen, J. (2019). Testing mechanisms underlying responses of plant functional traits to flooding duration gradient in a lakeshore meadow. Journal of Freshwater Ecology, 34(1), 481–495. 10.1080/02705060.2018.1550022

[ece311533-bib-0031] Li, F. , Gao, H. , Zhu, L. , Xie, Y. , Hu, C. , Chen, X. , & Deng, Z. (2017). Foliar nitrogen and phosphorus stoichiometry of three wetland plants distributed along an elevation gradient in Dongting Lake, China. Scientific Reports, 7, 2820. 10.1038/s41598-017-03126-9 28588236 PMC5460233

[ece311533-bib-0032] Li, J.‐E. , Nie, S.‐P. , Qiu, Z.‐H. , Che, M.‐J. , Li, C. , & Xie, M.‐Y. (2010). Antimicrobial and antioxidant activities of the essential oil from Herba Moslae. Journal of the Science of Food and Agriculture, 90(8), 1347–1352. 10.1002/jsfa.3941 20474054

[ece311533-bib-0033] Li, S. , Dong, S. , Shen, H. , Han, Y. , Zhang, J. , Xu, Y. , Gao, X. , Yang, M. , Li, Y. , Zhao, Z. , Liu, S. , Zhou, H. , Dong, Q. , & Jane, C. Y. (2019). Different responses of multif‐aceted plant diversities of alpine meadow and alpine steppe to nitrogen addition gradients on Qinghai‐Tibetan plateau. Science of the Total Environment, 688, 1405–1412. 10.1016/j.scitotenv.2019.06.211 31726568

[ece311533-bib-0034] Li, T. , Zhu, Z. , Shao, Y. , Chen, Z. , & Roß‐Nickoll, M. (2022). Soil seedbank: Importance for revegetation in the water level fluctuation zone of the reservoir area. Science of the Total Environment, 829, 154686. 10.1016/j.scitotenv.2022.154686 35314245

[ece311533-bib-0035] Li, X. , He, D. , Chen, G. , Yang, J. , Yang, Z. , Guo, X. J. , Wang, C. , Zhu, S. , Huang, Y. , Chen, H. , Huang, G. , Zhang, D. , & Ye, C. (2022). Responses of leaf functional traits to different hydrological regimes and leaf economics spectrum in the water level fluctuation zone of three gorges reservoir, China. Frontiers in Plant Science, 13, 1–15. 10.3389/fpls.2022.939452 PMC9478546 36119629

[ece311533-bib-0036] Li, Y. , Liu, C. , Xu, L. , Li, M. , Zhang, J. , & He, N. (2021). Leaf trait networks based on global data: Representing variation and adaptation in plants. Frontiers in Plant Science, 12, 1–10. 10.3389/fpls.2021.710530 PMC8688851 34950156

[ece311533-bib-0037] Liro, M. (2019). Dam reservoir backwater as a field‐scale laboratory of human‐induced changes in river biogeomorphology: A review focused on gravel‐bed rivers. Science of the Total Environment, 651, 2899–2912. 10.1016/j.scitotenv.2018.10.138 30463142

[ece311533-bib-0038] Liu, Z. , Zhao, M. , Zhang, H. , Ren, T. , Liu, C. , & He, N. (2023). Divergent response and adaptation of specific leaf area to environmental change at different spatiotemporal scales jointly improve plant survival. Global Change Biology, 29, 1144–1159. 10.1111/gcb.16518 36349544

[ece311533-bib-0039] Lu, H. (2019). Study on plant stoichiometry in the riparian zone of the Three Gorges Reservoir. Chongqing University. 10.27670/d.cnki.gcqdu.2019.001340

[ece311533-bib-0040] Nahar, L. , Nath, S. , & Sarker, S. D. (2022). “Malancha” [Alternanthera philoxeroides (Mart.) Griseb.]: A potential therapeutic option against viral diseases. Biomolecules, 12, 582. 10.3390/biom12040582 35454170 PMC9025398

[ece311533-bib-0041] Oettel, J. , Braun, M. , Sallmannshofer, M. , De Groot, M. , Schueler, S. , Virgillito, C. , & Lapin, K. (2022). River distance, stand basal area, and climatic conditions are the main drivers influencing lying deadwood in riparian forests. Forest Ecology and Management, 520, 120415. 10.1016/j.foreco.2022.120415

[ece311533-bib-0042] Pan, H. , Xiu, C. , Liu, B. , & Lu, Y. (2019). Plant stalks as oviposition traps for *Apolygus lucorum* (Hemiptera: Miridae) under field conditions. International Journal of Pest Management, 65, 79–85. 10.1080/09670874.2018.1462540

[ece311533-bib-0043] Procknow, D. , Rovedder, A. P. M. , Piaia, B. B. , Camargo, B. , de Moraes Stefanello, M. , da Silva, M. P. K. L. , & Dreyer, J. B. B. (2023). Monitoring ecological restoration of riparian forest: Is the applied nucleation effective ten years after implementation in the Pampa? Forest Ecology and Management, 538, 120955. 10.1016/j.foreco.2023.120955

[ece311533-bib-0044] Purcell, A. S. T. , Lee, W. G. , Tanentzap, A. J. , & Laughlin, D. C. (2019). Fine root traits are correlated with flooding duration while aboveground traits are related to grazing in an ephemeral wetland. Wetlands, 39(2), 291–302. 10.1007/s13157-018-1084-8

[ece311533-bib-0045] Rao, J. , Duan, D. , Tang, Q. , Ma, M. , Wei, J. , & He, X. (2023). Vegetation differentiation along elevation gradient in the water level fluctuation zone of the three gorges reservoir and its response to habitat stressing. Acta Ecologica Sinica, 43, 6649–6660.

[ece311533-bib-0046] Rees, T. A. V. , & Raven, J. A. (2021). The maximum growth rate hypothesis is correct for eukaryotic photosynthetic organisms, but not cyanobacteria. New Phytologist, 230, 601–611. 10.1111/nph.17190 33449358 PMC8048539

[ece311533-bib-0047] Su, X. , Bejarano, M. D. , Yi, X. , Lin, F. , Ayi, Q. , & Zeng, B. (2020). Unnatural flooding alters the functional diversity of riparian vegetation of the three gorges reservoir. Freshwater Biology, 65, 1585–1595. 10.1111/fwb.13523

[ece311533-bib-0074] Sun, M. , Tian, K. , Zhang, Y. , Wang, H. , Guan, D. X. , & Yue, H. T. (2017). Research on leaf functional traits and their environmental adaptation [J]. Plant Science Journal, 35(6): 940–949.

[ece311533-bib-0048] Sun, J. , Yuan, X. , Liu, H. , & Liu, G. (2021). Emergy and eco‐exergy evaluation of wetland reconstruction based on ecological engineering approaches in the three gorges reservoir, China. Ecological Indicators, 122, 107278. 10.1016/j.ecolind.2020.107278

[ece311533-bib-0049] Teng, M. , Huang, C. , Wang, P. , Zeng, L. , Zhou, Z. , Xiao, W. , Huang, Z. , & Liu, C. (2019). Impacts of forest restoration on soil erosion in the three gorges reservoir area, China. Science of the Total Environment, 697, 134164. 10.1016/j.scitotenv.2019.134164 32380623

[ece311533-bib-0050] Tian, D. , Yan, Z.‐B. , & Fang, J.‐Y. (2021). Review on characteristics and main hypotheses of plant ecological stoichiometry. Chinese Journal of Plant Ecology, 45, 682–713. 10.17521/cjpe.2020.0331

[ece311533-bib-0051] Toner, M. , & Keddy, P. (1997). River hydrology and riparian wetlands: A predictive model for ecological assembly. Ecological Applications, 7(1), 236–246. 10.1890/1051-0761(1997)007[0236:RHARWA]2.0.CO;2

[ece311533-bib-0052] Vesipa, R. , Camporeale, C. , & Ridolfi, L. (2016). Recovery times of riparian vegetation. Water Resources Research, 52(4), 2934–2950. 10.1002/2015WR018490

[ece311533-bib-0053] Wang, C. , Li, X. , Lu, X. , Wang, Y. , & Bai, Y. (2023). Intraspecific trait variation governs grazing‐induced shifts in plant community above‐and below‐ground functional trait composition. Agriculture, Ecosystems & Environment, 346, 108357. 10.1016/j.agee.2023.108357

[ece311533-bib-0054] Wang, H. , Sun, T. , Liu, Y. , & Xiao, H. (2023). Stoichiometric characteristics and homeostasis of leaf nitrogen and phosphorus responding to different water surface elevations in hydro‐fluctuation zone of the three gorges reservoir. Aquatic Sciences, 85, 80. 10.1007/s00027-023-00977-5

[ece311533-bib-0056] Wang, M. , Wan, P. , Guo, J. , Xu, J. , Chai, Y. , & Yue, M. (2017). Relationships among leaf, stem and root traits of the dominant shrubs from four vegetation zones in Shaanxi Province, China. Israel Journal of Ecology & Evolution, 63, 25–32. 10.1163/22244662-06301005

[ece311533-bib-0075] Wang, Q. (2020). Leaf traits of 110 landscape plant species in Nanchang. [D]. Jiangxi Normal University.

[ece311533-bib-0057] Wang, Z. , & Wang, C. (2023). Individual and interactive responses of woody plants' biomass and leaf traits to drought and shade. Global Ecology and Biogeography, 32, 35–48. 10.1111/geb.13615

[ece311533-bib-0058] Wei, Z. , Halik, Ü. , Aishan, T. , Abliz, A. , & Welp, M. (2022). Spatial distribution patterns of trunk internal decay of *Euphrates poplar* riparian forest along the Tarim River, northwest China. Forest Ecology and Management, 522, 120434. 10.1016/j.foreco.2022.120434

[ece311533-bib-0059] Willison, J. H. M. , Li, R. , & Yuan, X. (2013). Conservation and ecofriendly utilization of wetlands associated with the three gorges reservoir. Environmental Science and Pollution Research, 20, 6907–6916. 10.1007/s11356-012-1438-3 23288679

[ece311533-bib-0076] Xia, D. J. , Liu, Q. R. , Zou, L. L. , Ge, Z. W. , Xue, J. H. , & Peng, S. L. (2020). Foliar δ13C correlates with elemental stoichiometry in halophytes of coastal wetlands. [J]. Acta Ecologica Sinica, 2020, 40(7): 2215–2224.

[ece311533-bib-0060] Xuan, T. D. , Tsuzuki, E. , Hiroyuki, T. , Mitsuhiro, M. , Khanh, T. D. , & Chung, I.‐M. (2004). Evaluation on phytotoxicity of neem (*Azadirachta indica*. A. Juss) to crops and weeds. Crop Protection, 23(4), 335–345. 10.1016/j.cropro.2003.09.004

[ece311533-bib-0061] Yan, Y. , Liu, Q. , Zhang, Q. , Ding, Y. , & Li, Y. (2019). Adaptation of dominant species to drought in the Inner Mongolia grassland‐species level and functional type level analysis. Frontiers in Plant Science, 10, 1–10. 10.3389/fpls.2019.00231 31040855 PMC6477032

[ece311533-bib-0062] Ye, C. , Butler, O. M. , Chen, C. , Liu, W. , Du, M. , & Zhang, Q. (2020). Shifts in characteristics of the plant‐soil system associated with flooding and revegetation in the riparian zone of three gorges reservoir, China. Geoderma, 361, 114015. 10.1016/j.geoderma.2019.114015

[ece311533-bib-0077] You, G. H. , Wang, Y. L. , & Wang, C. T. (2022). Response of plant leaf ecological stoichiometric characteristics to long‐term nitrogen addition in alpine meadow. Acta Prataculturae Sinica, 31(9): 50–62.

[ece311533-bib-0063] Zhang, A. , & Xie, Z. (2021). C4 herbs dominate the reservoir flood area of the three gorges reservoir. Science of the Total Environment, 755, 142479. 10.1016/j.scitotenv.2020.142479 33035969

[ece311533-bib-0064] Zhang, D. , Qi, Q. , Wang, X. , Tong, S. , Lv, X. , An, Y. , & Zhu, X. (2019). Physiological responses of *Carex schmidtii* Meinsh to alternating flooding‐drought conditions in the Momoge wetland, northeast China. Aquatic Botany, 153, 33–39. 10.1016/j.aquabot.2018.11.010

[ece311533-bib-0065] Zhang, J. , Saqib, H. S. A. , Niu, D. , Guaman, K. G. G. , Wang, A. , Yu, D. , You, M. , Pozsgai, G. , & You, S. (2023). Contrasting roles of landscape compositions on shaping functional traits of arthropod community in subtropical vegetable fields. Agriculture, Ecosystems & Environment, 347, 108386. 10.1016/j.agee.2023.108386

[ece311533-bib-0066] Zhang, X. , Fang, C. , Wang, Y. , Lou, X. , Su, Y. , & Huang, D. (2022). Review of effects of dam construction on the ecosystems of river estuary and nearby marine areas. Sustainability, 14, 5974. 10.3390/su14105974

[ece311533-bib-0067] Zhao, Y. , Sun, Y. , Lu, X. , Zhao, X. , Yang, L. , Sun, Z. , & Bai, Y. (2021). Hyperspectral retrieval of leaf physiological traits and their links to ecosystem productivity in grass‐land monocultures. Ecological Indicators, 122, 107267. 10.1016/j.ecolind.2020.107267

[ece311533-bib-0068] Zheng, J. , Arif, M. , He, X. , Liu, X. , & Li, C. (2023). Distinguishing the mechanisms driving multifaceted plant diversity in subtropical reservoir riparian zones. Frontiers in Plant Science, 14, 11383. 10.3389/fpls.2023.1138368 PMC9998900 36909398

[ece311533-bib-0069] Zheng, J. , Arif, M. , Zhang, S. , Yuan, Z. , Zhang, L. , Dong, Z. , Tan, X. , Charles, W. , & Li, C. (2021). The convergence of species composition along the drawdown zone of the three gorges dam reservoir, China: Implications for restoration. Environmental Science and Pollution Research, 28, 42609–42621. 10.1007/s11356-021-13774-0 33818726

[ece311533-bib-0070] Zheng, J. , Arif, M. , Zhang, S. , Yuan, Z. , Zhang, L. , Li, J. , Ding, D. , & Li, C. (2021). Dam inundation simplifies the plant community composition. Science of the Total Environment, 801, 149827. 10.1016/j.scitotenv.2021.149827 34467924

[ece311533-bib-0071] Zhu, K.‐W. , Chen, Y.‐C. , Zhang, S. , Lei, B. , Yang, Z.‐M. , & Huang, L. (2020). Vegetation of the water‐level fluctuation zone in the three gorges reservoir at the initial impoundment stage. Global Ecology and Conservation, 21, e00866. 10.1016/j.gecco.2019.e00866

[ece311533-bib-0072] Zhu, Z. , Chen, Z. , Li, L. , & Shao, Y. (2020). Response of dominant plant species to periodic flooding in the riparian zone of the three gorges reservoir (TGR), China. Science of the Total Environment, 747, 141101. 10.1016/j.scitotenv.2020.141101 32771779

[ece311533-bib-0073] Zhu, Z. , Wu, S. , Wang, Y. , Wang, J. , & Zhang, Y. (2022). Reveal the antimigraine mechanism of chuanxiong Rhizoma and Cyperi Rhizoma based on the integrated analysis of metabolomics and network pharmacology. Frontiers in Pharmacology, 13, 1–14. 10.3389/fphar.2022.805984 PMC8987590 35401159

