# Supplementary material for: Assessing leaf physiological traits in response to flooding among dominant riparian herbs along the Three Gorges Dam in China
Source: Ecol Evol. 2024 Jun 21;14(6):e11533. doi: 10.1002/ece3.11533 (PMC11192621; doi:10.1002/ece3.11533)
Supplement: Supplementary file 2 — Data S2. [file ECE3-14-e11533-s002.docx]

# Article related key analysis data code

**cluster analysis**

library(ape)

hc2<-hclust(dist(log10(df2[,2:7])),method = "ward.D2")

par(font=4,ps=4)

hc2$labels<-paste(df2$species)

plot(hc2)

clus5<-cutree(hc2,3)

h3<-c('salmon','seagreen','dodgerblue')

plot(as.phylo(hc),tip.color=h1[clus5],label.offset = 0.01,cex=2)

**Multiple comparisons between different functional types**

library(car)

library(PMCMRplus)

library(agricolae)

element<-read.csv('li2.csv',header = TRUE)

leveneTest(element$LCC,element$altitude)

shapiro.test(element$LCC)

kruskal.test(LCC~altitude,data = element)

co1<-with(element,kruskal(LCC,altitude,p.adj='bon',group = T))

co1

f<-aov(LCC~altitude,data = element)

summary(f)

re8<-LSD.test(f,'altitude',alpha = 0.01)

re9<-re8$groups

re9

leveneTest(element$LNC,element$altitude)

shapiro.test(element$LNC)

kruskal.test(LNC~altitude,data = element)

co1<-with(element,kruskal(LNC,altitude,p.adj='bon',group = T))

co1

leveneTest(element$LPC,element$altitude)

shapiro.test(element$LPC)

kruskal.test(LPC~altitude,data = element)

co1<-with(element,kruskal(LPC,altitude,p.adj='bon',group = T))

co1

leveneTest(element$CNR,element$altitude)

shapiro.test(element$CNR)

kruskal.test(CNR~altitude,data = element)

co1<-with(element,kruskal(CNR,altitude,p.adj='bon',group = T))

co1

leveneTest(element$CPR,element$altitude)

shapiro.test(element$CPR)

kruskal.test(CPR~altitude,data = element)

co1<-with(element,kruskal(CPR,altitude,p.adj='bon',group = T))

co1

leveneTest(element$NPR,element$altitude)

shapiro.test(element$NPR)

kruskal.test(NPR~altitude,data = element)

co1<-with(element,kruskal(NPR,altitude,p.adj='bon',group = T))

co1

leveneTest(element$LCC,element$lifeform)

shapiro.test(element$LCC)

kruskal.test(LCC~lifeform,data = element)

co1<-with(element,kruskal(LCC,lifeform,p.adj='bon',group = T))

co1

leveneTest(element$LNC,element$lifeform)

shapiro.test(element$LNC)

kruskal.test(LNC~lifeform,data = element)

co1<-with(element,kruskal(LNC,lifeform,p.adj='bon',group = T))

co1

leveneTest(element$LPC,element$lifeform)

shapiro.test(element$LPC)

kruskal.test(LPC~lifeform,data = element)

co1<-with(element,kruskal(LPC,lifeform,p.adj='bon',group = T))

co1

leveneTest(element$CNR,element$lifeform)

shapiro.test(element$CNR)

kruskal.test(CNR~lifeform,data = element)

co1<-with(element,kruskal(CNR,lifeform,p.adj='bon',group = T))

co1

leveneTest(element$CPR,element$lifeform)

shapiro.test(element$CPR)

kruskal.test(CPR~lifeform,data = element)

co1<-with(element,kruskal(CPR,lifeform,p.adj='bon',group = T))

co1

leveneTest(element$NPR,element$lifeform)

shapiro.test(element$NPR)

kruskal.test(NPR~lifeform,data = element)

co1<-with(element,kruskal(NPR,lifeform,p.adj='bon',group = T))

co1

element<-read.csv('li6.csv',header = TRUE)

leveneTest(element$LCC,element$lifeform)

shapiro.test(element$LCC)

kruskal.test(LCC~lifeform,data = element)

co1<-with(element,kruskal(LCC,lifeform,p.adj='bon',group = T))

co1

f<-aov(LCC~lifeform,data = element)

summary(f)

re8<-LSD.test(f,'lifeform',alpha = 0.01)

re9<-re8$groups

re9

leveneTest(element$LNC,element$lifeform)

shapiro.test(element$LNC)

kruskal.test(LNC~lifeform,data = element)

co1<-with(element,kruskal(LNC,lifeform,p.adj='bon',group = T))

co1

f<-aov(LCC~lifeform,data = element)

summary(f)

re8<-LSD.test(f,'lifeform',alpha = 0.01)

re9<-re8$groups

re9

leveneTest(element$LPC,element$lifeform)

shapiro.test(element$LPC)

kruskal.test(LPC~lifeform,data = element)

co1<-with(element,kruskal(LPC,lifeform,p.adj='bon',group = T))

co1

f<-aov(LCC~lifeform,data = element)

summary(f)

re8<-LSD.test(f,'lifeform',alpha = 0.01)

re9<-re8$groups

re9

leveneTest(element$CNR,element$lifeform)

shapiro.test(element$CNR)

kruskal.test(CNR~lifeform,data = element)

co1<-with(element,kruskal(CNR,lifeform,p.adj='bon',group = T))

co1

f<-aov(LCC~lifeform,data = element)

summary(f)

re8<-LSD.test(f,'lifeform',alpha = 0.01)

re9<-re8$groups

re9

leveneTest(element$CPR,element$lifeform)

shapiro.test(element$CPR)

kruskal.test(CPR~lifeform,data = element)

co1<-with(element,kruskal(CPR,lifeform,p.adj='bon',group = T))

co1

f<-aov(LCC~lifeform,data = element)

summary(f)

re8<-LSD.test(f,'lifeform',alpha = 0.01)

re9<-re8$groups

re9

leveneTest(element$NPR,element$lifeform)

shapiro.test(element$NPR)

kruskal.test(NPR~lifeform,data = element)

co1<-with(element,kruskal(NPR,lifeform,p.adj='bon',group = T))

co1

f<-aov(NPR~lifeform,data = element)

summary(f)

re8<-LSD.test(f,'lifeform',alpha = 0.01)

re9<-re8$groups

re9

leveneTest(element$LNC,element$altitude)

shapiro.test(element$LNC)

kruskal.test(LNC~altitude,data = element)

co1<-with(element,kruskal(LNC,altitude,p.adj='bon',group = T))

co1

leveneTest(element$LPC,element$altitude)

shapiro.test(element$LPC)

kruskal.test(LPC~altitude,data = element)

co1<-with(element,kruskal(LPC,altitude,p.adj='bon',group = T))

co1

leveneTest(element$CNR,element$altitude)

shapiro.test(element$CNR)

kruskal.test(CNR~altitude,data = element)

co1<-with(element,kruskal(CNR,altitude,p.adj='bon',group = T))

co1

leveneTest(element$CPR,element$altitude)

shapiro.test(element$CPR)

kruskal.test(CPR~altitude,data = element)

co1<-with(element,kruskal(CPR,altitude,p.adj='bon',group = T))

co1

leveneTest(element$NPR,element$altitude)

shapiro.test(element$NPR)

kruskal.test(NPR~altitude,data = element)

co1<-with(element,kruskal(NPR,altitude,p.adj='bon',group = T))

co1

leveneTest(element$LCC,element$lifeform)

shapiro.test(element$LCC)

kruskal.test(LCC~lifeform,data = element)

co1<-with(element,kruskal(LCC,lifeform,p.adj='bon',group = T))

co1

leveneTest(element$LNC,element$lifeform)

shapiro.test(element$LNC)

kruskal.test(LNC~lifeform,data = element)

co1<-with(element,kruskal(LNC,lifeform,p.adj='bon',group = T))

co1

leveneTest(element$LPC,element$lifeform)

shapiro.test(element$LPC)

kruskal.test(LPC~lifeform,data = element)

co1<-with(element,kruskal(LPC,lifeform,p.adj='bon',group = T))

co1

leveneTest(element$CNR,element$lifeform)

shapiro.test(element$CNR)

kruskal.test(CNR~lifeform,data = element)

co1<-with(element,kruskal(CNR,lifeform,p.adj='bon',group = T))

co1

leveneTest(element$CPR,element$lifeform)

shapiro.test(element$CPR)

kruskal.test(CPR~lifeform,data = element)

co1<-with(element,kruskal(CPR,lifeform,p.adj='bon',group = T))

co1

leveneTest(element$NPR,element$lifeform)

shapiro.test(element$NPR)

kruskal.test(NPR~lifeform,data = element)

co1<-with(element,kruskal(NPR,lifeform,p.adj='bon',group = T))

co1

leveneTest(element$LNC,element$type)

shapiro.test(element$LNC)

f<-aov(LNC~type,data = element)

summary(f)

re8<-LSD.test(f,'type',alpha = 0.01)

re9<-re8$groups

re9

kruskal.test(LNC~type,data = element)

co2<-with(element,kruskal(LNC,type,p.adj='bon',group = T))

co2

kruskalTest(element$LNC~element$type)

kwAllPairsDunnTest(element$LNC~element$type,element,p.adjust.method = 'bonferroni')

leveneTest(element$LPC,element$type)

shapiro.test(element$LPC)

a<-aov(LPC~type,data = element)

summary(a)

kruskal.test(LPC~type,data = element)

co3<-with(element,kruskal(LPC,type,p.adj='bon',group = T))

co3

re<-LSD.test(a,'type',alpha = 0.01)

re1<-re$groups

re1

leveneTest(element$CNR,element$type)

shapiro.test(element$CNR)

b<-aov(CNR~type,data = element)

summary(b)

re2<-LSD.test(b,'type',alpha = 0.01)

re3<-re2$groups

re3

kruskal.test(CNR~type,data = element)

co5<-with(element,kruskal(CNR,type,p.adj='bon',group = T))

co5

leveneTest(element$CPR,element$type)

shapiro.test(element$CPR)

kruskal.test(CPR~type,data = element)

co4<-with(element,kruskal(CPR,type,p.adj='bon',group = T))

co4

d<-aov(CPR~type,data = element)

summary(d)

re4<-LSD.test(d,'type',alpha = 0.01)

re5<-re4$groups

re5

leveneTest(element$NPR,element$type)#方差齐性检验

shapiro.test(element$NPR)#正态检验

e<-aov(NPR~type,data = element)

summary(e)

re6<-LSD.test(e,'type',alpha = 0.01)#单因素方差分析的多重比较

re7<-re6$groups

re7

kruskal.test(NPR~type,data = element)#非参数检验

co6<-with(element,kruskal(NPR,type,p.adj='bon',group = T))#非参数多重比较

co6

library(car)

library(PMCMRplus)

library(agricolae)

leveneTest(element$LCC,element$altitude)

shapiro.test(element$LCC)

kruskal.test(LCC~altitude,data = element)

co1<-with(element,kruskal(LCC,altitude,p.adj='bon',group = T))

co1

g<-aov(LCC~altitude,data = element)

summary(g)

re10<-LSD.test(g,'altitude',alpha = 0.01)

re11<-re10$groups

re11

leveneTest(element$LNC,element$altitude)

shapiro.test(element$LNC)

f<-aov(LNC~altitude,data = element)

summary(f)

re8<-LSD.test(f,'altitude',alpha = 0.01)

re9<-re8$groups

re9

kruskal.test(LNC~altitude,data = element)

co2<-with(element,kruskal(LNC,altitude,p.adj='bon',group = T))

co2

leveneTest(element$LPC,element$altitude)

shapiro.test(element$LPC)

a<-aov(LPC~altitude,data = element)

summary(a)

kruskal.test(LPC~altitude,data = element)

co3<-with(element,kruskal(LPC,altitude,p.adj='bon',group = T))

co3

re<-LSD.test(a,'altitude',alpha = 0.01)

re1<-re$groups

re1

leveneTest(element$CNR,element$altitude)

shapiro.test(element$CNR)

b<-aov(CNR~altitude,data = element)

summary(b)

re2<-LSD.test(b,'altitude',alpha = 0.01)

re3<-re2$groups

re3

kruskal.test(CNR~altitude,data = element)

co5<-with(element,kruskal(CNR,altitude,p.adj='bon',group = T))

co5

leveneTest(element$CPR,element$altitude)

shapiro.test(element$CPR)

kruskal.test(CPR~altitude,data = element)

co4<-with(element,kruskal(CPR,altitude,p.adj='bon',group = T))

co4

d<-aov(CPR~altitude,data = element)

summary(d)

re4<-LSD.test(d,'altitude',alpha = 0.01)

re5<-re4$groups

re5

leveneTest(element$NPR,element$altitude)#方差齐性检验

shapiro.test(element$NPR)#正态检验

e<-aov(NPR~altitude,data = element)

summary(e)

re6<-LSD.test(e,'altitude',alpha = 0.01)#单因素方差分析的多重比较

re7<-re6$groups

re7

kruskal.test(NPR~altitude,data = element)#非参数检验

co6<-with(element,kruskal(NPR,altitude,p.adj='bon',group = T))#非参数多重比较

co6

**Visualize graphics in the ggplot2 package**

library(ggplot2)

library(gcookbook)

library(patchwork)

library(ggpubr)

df<-element

p1<-ggplot(data = df,aes(x=Altitude,y=LCC,fill=PFTs))+

stat_boxplot(geom = 'errorbar',

width=0.3,

position = position_dodge(0.8))+

geom_boxplot(position = position_dodge(0.8))+

theme_bw()+

ylab("LCC(%)")+

xlab(NULL)+

theme(axis.title.y= element_text(size = 10,family='serif'))+

theme(axis.text.y=element_text(size = 7))+

annotate('text',x=0.72,y=44,label='Aa',family='serif',

size=4,color='red')+

annotate('text',x=1,y=41.3,label='Aa',family='serif',

size=4,color='red')+

annotate('text',x=1.29,y=43.3,label='ABa',family='serif',

size=4,color='red')+

annotate('text',x=1.72,y=45.7,label='Aa',family='serif',

size=4,color='red')+

annotate('text',x=2,y=40.7,label='Aa',family='serif',

size=4,color='red')+

annotate('text',x=2.25,y=42.7,label='Ba',family='serif',

size=4,color='red')+

annotate('text',x=2.72,y=44.7,label='Aa',family='serif',

size=4,color='red')+

annotate('text',x=3,y=43.8,label='Aa',family='serif',

size=4,color='red')+

annotate('text',x=3.28,y=46,label='ABa',family='serif',

size=4,color='red')+

annotate('text',x=3.73,y=45.4,label='Aa',family='serif',

size=4,color='red')+

annotate('text',x=4,y=43.3,label='Aa',family='serif',

size=4,color='red')+

annotate('text',x=4.27,y=48,label='Aa',family='serif',

size=4,color='red')

p2<-ggplot(data = df,aes(x=Altitude,y=LNC,fill=PFTs))+

stat_boxplot(geom = 'errorbar',

width=0.3,

position = position_dodge(0.8))+

geom_boxplot(position = position_dodge(0.8))+

theme_bw()+

ylab("LNC(%)")+

xlab(NULL)+

theme(axis.title.y= element_text(size = 10,family='serif'))+

theme(axis.text.y=element_text(size = 7))+

annotate('text',x=0.72,y=2.06,label='Ab',family='serif',

size=4,color='red')+

annotate('text',x=1,y=1.95,label='Ab',family='serif',

size=4,color='red')+

annotate('text',x=1.25,y=5.76,label='Aa',family='serif',

size=4,color='red')+

annotate('text',x=1.73,y=2.4,label='Aa',family='serif',

size=4,color='red')+

annotate('text',x=2,y=1.5,label='Aa',family='serif',

size=4,color='red')+

annotate('text',x=2.25,y=4.25,label='Aa',family='serif',

size=4,color='red')+

annotate('text',x=2.72,y=2.25,label='Ab',family='serif',

size=4,color='red')+

annotate('text',x=3,y=1.6,label='Ab',family='serif',

size=4,color='red')+

annotate('text',x=3.28,y=4.75,label='Aa',family='serif',

size=4,color='red')+

annotate('text',x=3.73,y=2.65,label='Ab',family='serif',

size=4,color='red')+

annotate('text',x=4,y=1.35,label='Ac',family='serif',

size=4,color='red')+

annotate('text',x=4.25,y=5.05,label='Aa',family='serif',

size=4,color='red')

p3<-ggplot(data = df,aes(x=Altitude,y=LPC,fill=PFTs))+

stat_boxplot(geom = 'errorbar',

width=0.3,

position = position_dodge(0.8))+

geom_boxplot(position = position_dodge(0.8))+

theme_bw()+

ylab("LPC(%)")+

xlab(NULL)+

theme(axis.title.y= element_text(size = 10,family='serif'))+

theme(axis.text.y=element_text(size = 7))+

annotate('text',x=0.72,y=0.33,label='Ab',family='serif',

size=4,color='red')+

annotate('text',x=1,y=1.04,label='Aa',family='serif',

size=4,color='red')+

annotate('text',x=1.28,y=1.11,label='Aa',family='serif',

size=4,color='red')+

annotate('text',x=1.73,y=0.48,label='Ab',family='serif',

size=4,color='red')+

annotate('text',x=2,y=0.76,label='Ab',family='serif',

size=4,color='red')+

annotate('text',x=2.25,y=1.56,label='Aa',family='serif',

size=4,color='red')+

annotate('text',x=2.72,y=0.55,label='Ac',family='serif',

size=4,color='red')+

annotate('text',x=3,y=0.84,label='Ab',family='serif',

size=4,color='red')+

annotate('text',x=3.28,y=1.45,label='Aa',family='serif',

size=4,color='red')+

annotate('text',x=3.73,y=0.52,label='Ac',family='serif',

size=4,color='red')+

annotate('text',x=4,y=1.09,label='Ab',family='serif',

size=4,color='red')+

annotate('text',x=4.25,y=1.35,label='Aa',family='serif',

size=4,color='red')

p4<-ggplot(data = df,aes(x=Altitude,y=CNR,fill=PFTs))+

stat_boxplot(geom = 'errorbar',

width=0.3,

position = position_dodge(0.8))+

geom_boxplot(position = position_dodge(0.8))+

theme_bw()+

ylab("LC:N")+

xlab(NULL)+

theme(axis.title.y= element_text(size = 10,family='serif'))+

theme(axis.text.y=element_text(size = 7))+

annotate('text',x=0.72,y=56,label='Aa',family='serif',

size=4,color='red')+

annotate('text',x=1,y=56,label='Aa',family='serif',

size=4,color='red')+

annotate('text',x=1.25,y=44.5,label='Aab',family='serif',

size=4,color='red')+

annotate('text',x=1.73,y=52.5,label='Aa',family='serif',

size=4,color='red')+

annotate('text',x=2,y=42.5,label='Aa',family='serif',

size=4,color='red')+

annotate('text',x=2.25,y=33.5,label='Aa',family='serif',

size=4,color='red')+

annotate('text',x=2.72,y=59.5,label='Aa',family='serif',

size=4,color='red')+

annotate('text',x=3,y=81,label='Aa',family='serif',

size=4,color='red')+

annotate('text',x=3.28,y=24.5,label='Ab',family='serif',

size=4,color='red')+

annotate('text',x=3.73,y=56,label='Aa',family='serif',

size=4,color='red')+

annotate('text',x=4,y=67.5,label='Ab',family='serif',

size=4,color='red')+

annotate('text',x=4.25,y=22.5,label='Ac',family='serif',

size=4,color='red')

p5<-ggplot(data = df,aes(x=Altitude,y=CPR,fill=PFTs))+

stat_boxplot(geom = 'errorbar',

width=0.3,

position = position_dodge(0.8))+

geom_boxplot(position = position_dodge(0.8))+

theme_bw()+

ylab("LC:P")+

theme(axis.title.y= element_text(size = 10,family='serif'))+

theme(axis.text.y=element_text(size = 7))+

annotate('text',x=0.72,y=540,label='Aa',family='serif',

size=4,color='red')+

annotate('text',x=1,y=100,label='Ab',family='serif',

size=4,color='red')+

annotate('text',x=1.25,y=140,label='Ab',family='serif',

size=4,color='red')+

annotate('text',x=1.73,y=490,label='Aa',family='serif',

size=4,color='red')+

annotate('text',x=2,y=200,label='Ab',family='serif',

size=4,color='red')+

annotate('text',x=2.25,y=110,label='Ab',family='serif',

size=4,color='red')+

annotate('text',x=2.72,y=680,label='Aa',family='serif',

size=4,color='red')+

annotate('text',x=3,y=330,label='Ab',family='serif',

size=4,color='red')+

annotate('text',x=3.28,y=150,label='Ac',family='serif',

size=4,color='red')+

annotate('text',x=3.73,y=440,label='Aa',family='serif',

size=4,color='red')+

annotate('text',x=4,y=310,label='Ab',family='serif',

size=4,color='red')+

annotate('text',x=4.25,y=140,label='Ac',family='serif',

size=4,color='red')

p6<-ggplot(data = df,aes(x=Altitude,y=NPR,fill=PFTs))+

stat_boxplot(geom = 'errorbar',

width=0.3,

position = position_dodge(0.8))+

geom_boxplot(position = position_dodge(0.8))+

theme_bw()+

ylab("LN:P")+

theme(axis.title.y= element_text(size = 10,family='serif'))+

theme(axis.text.y=element_text(size = 7))+

annotate('text',x=0.72,y=16.9,label='Aa',family='serif',

size=4,color='red')+

annotate('text',x=1,y=3.1,label='Ab',family='serif',

size=4,color='red')+

annotate('text',x=1.25,y=8,label='ABb',family='serif',

size=4,color='red')+

annotate('text',x=1.73,y=15.3,label='Aa',family='serif',

size=4,color='red')+

annotate('text',x=2,y=5.9,label='Ab',family='serif',

size=4,color='red')+

annotate('text',x=2.258,y=5.5,label='Bb',family='serif',

size=4,color='red')+

annotate('text',x=2.72,y=26.1,label='Aa',family='serif',

size=4,color='red')+

annotate('text',x=3,y=6.5,label='Ab',family='serif',

size=4,color='red')+

annotate('text',x=3.28,y=8.5,label='ABb',family='serif',

size=4,color='red')+

annotate('text',x=3.75,y=13.5,label='Aa',family='serif',

size=4,color='red')+

annotate('text',x=4,y=6.5,label='Ab',family='serif',

size=4,color='red')+

annotate('text',x=4.25,y=10.1,label='Ab',family='serif',

size=4,color='red')

ggarrange(p1,p2,p3,p4,p5,p6,ncol = 2,nrow = 3,common.legend = TRUE,legend = 'right',

labels = c('A','B','C','D','E','F'),

font.label = list(size=10,family='serif',color='brown'))

df1<-lifeform

p11<-ggplot(data = df1,aes(x=altitude,y=LCC,fill=lifeform))+

stat_boxplot(geom = 'errorbar',

width=0.3,

position = position_dodge(0.8))+

geom_boxplot(position = position_dodge(0.8))+

theme_bw()+

ylab("LCC(%)")+

xlab(NULL)+

theme(axis.title.y= element_text(size = 10,family='serif'))+

theme(axis.text.y=element_text(size = 7))+

annotate('text',x=0.8,y=44,label='Ba',family='serif',

size=4,color='red')+

annotate('text',x=1.2,y=42.5,label='Ba',family='serif',

size=4,color='red')+

annotate('text',x=1.8,y=45.5,label='ABa',family='serif',

size=4,color='red')+

annotate('text',x=2.2,y=43.5,label='ABa',family='serif',

size=4,color='red')+

annotate('text',x=2.8,y=45,label='ABa',family='serif',

size=4,color='red')+

annotate('text',x=3.2,y=45,label='Aa',family='serif',

size=4,color='red')+

annotate('text',x=3.8,y=46.5,label='Aa',family='serif',

size=4,color='red')+

annotate('text',x=4.2,y=45.5,label='Aa',family='serif',

size=4,color='red')

p22<-ggplot(data = df1,aes(x=altitude,y=LNC,fill=lifeform))+

stat_boxplot(geom = 'errorbar',

width=0.3,

position = position_dodge(0.8))+

geom_boxplot(position = position_dodge(0.8))+

theme_bw()+

ylab("LNC(%)")+

xlab(NULL)+

theme(axis.title.y= element_text(size = 10,family='serif'))+

theme(axis.text.y=element_text(size = 7))+

annotate('text',x=0.8,y=5.8,label='Aa',family='serif',

size=4,color='red')+

annotate('text',x=1.2,y=1.85,label='Ab',family='serif',

size=4,color='red')+

annotate('text',x=1.8,y=5.3,label='Aa',family='serif',

size=4,color='red')+

annotate('text',x=2.2,y=2.15,label='Ab',family='serif',

size=4,color='red')+

annotate('text',x=2.8,y=5,label='Aa',family='serif',

size=4,color='red')+

annotate('text',x=3.2,y=4.1,label='Aa',family='serif',

size=4,color='red')+

annotate('text',x=3.8,y=4.1,label='Aa',family='serif',

size=4,color='red')+

annotate('text',x=4.2,y=3.7,label='Aa',family='serif',

size=4,color='red')

p33<-ggplot(data = df1,aes(x=altitude,y=LPC,fill=lifeform))+

stat_boxplot(geom = 'errorbar',

width=0.3,

position = position_dodge(0.8))+

geom_boxplot(position = position_dodge(0.8))+

theme_bw()+

ylab("LPC(%)")+

xlab(NULL)+

theme(axis.title.y= element_text(size = 10,family='serif'))+

theme(axis.text.y=element_text(size = 7))+

annotate('text',x=0.8,y=1.32,label='Aa',family='serif',

size=4,color='red')+

annotate('text',x=1.2,y=0.42,label='Bb',family='serif',

size=4,color='red')+

annotate('text',x=1.8,y=1.31,label='Aa',family='serif',

size=4,color='red')+

annotate('text',x=2.2,y=0.49,label='Bb',family='serif',

size=4,color='red')+

annotate('text',x=2.8,y=1.44,label='Aa',family='serif',

size=4,color='red')+

annotate('text',x=3.2,y=1.14,label='Ab',family='serif',

size=4,color='red')+

annotate('text',x=3.8,y=1.03,label='Aa',family='serif',

size=4,color='red')+

annotate('text',x=4.2,y=0.67,label='Ab',family='serif',

size=4,color='red')

p44<-ggplot(data = df1,aes(x=altitude,y=CNR,fill=lifeform))+

stat_boxplot(geom = 'errorbar',

width=0.3,

position = position_dodge(0.8))+

geom_boxplot(position = position_dodge(0.8))+

theme_bw()+

ylab("LC:N")+

xlab(NULL)+

theme(axis.title.y= element_text(size = 10,family='serif'))+

theme(axis.text.y=element_text(size = 7))+

annotate('text',x=0.8,y=63.3,label='Ab',family='serif',

size=4,color='red')+

annotate('text',x=1.2,y=44,label='Aa',family='serif',

size=4,color='red')+

annotate('text',x=1.8,y=41.2,label='Ab',family='serif',

size=4,color='red')+

annotate('text',x=2.2,y=54.5,label='Aa',family='serif',

size=4,color='red')+

annotate('text',x=2.8,y=68.5,label='Aa',family='serif',

size=4,color='red')+

annotate('text',x=3.2,y=67.5,label='Aa',family='serif',

size=4,color='red')+

annotate('text',x=3.8,y=35.5,label='Aa',family='serif',

size=4,color='red')+

annotate('text',x=4.2,y=59.5,label='Aa',family='serif',

size=4,color='red')

p55<-ggplot(data = df1,aes(x=altitude,y=CPR,fill=lifeform))+

stat_boxplot(geom = 'errorbar',

width=0.3,

position = position_dodge(0.8))+

geom_boxplot(position = position_dodge(0.8))+

theme_bw()+

ylab("LC:P")+

theme(axis.title.y= element_text(size = 10,family='serif'))+

theme(axis.text.y=element_text(size = 7))+

annotate('text',x=0.8,y=180,label='Ab',family='serif',

size=4,color='red')+

annotate('text',x=1.2,y=540,label='Aa',family='serif',

size=4,color='red')+

annotate('text',x=1.8,y=140,label='Ab',family='serif',

size=4,color='red')+

annotate('text',x=2.2,y=670,label='Aa',family='serif',

size=4,color='red')+

annotate('text',x=2.8,y=150,label='Ab',family='serif',

size=4,color='red')+

annotate('text',x=3.2,y=550,label='Ba',family='serif',

size=4,color='red')+

annotate('text',x=3.8,y=140,label='Ab',family='serif',

size=4,color='red')+

annotate('text',x=4.2,y=430,label='Ba',family='serif',

size=4,color='red')

p66<-ggplot(data = df1,aes(x=altitude,y=NPR,fill=lifeform))+

stat_boxplot(geom = 'errorbar',

width=0.3,

position = position_dodge(0.8))+

geom_boxplot(position = position_dodge(0.8))+

theme_bw()+

ylab("LN:P")+

theme(axis.title.y= element_text(size = 10,family='serif'))+

theme(axis.text.y=element_text(size = 7))+

annotate('text',x=0.8,y=8,label='Ab',family='serif',

size=4,color='red')+

annotate('text',x=1.2,y=18.8,label='ABa',family='serif',

size=4,color='red')+

annotate('text',x=1.8,y=8,label='Ab',family='serif',

size=4,color='red')+

annotate('text',x=2.2,y=18.7,label='Aa',family='serif',

size=4,color='red')+

annotate('text',x=2.8,y=8.2,label='Ab',family='serif',

size=4,color='red')+

annotate('text',x=3.2,y=14.7,label='Ba',family='serif',

size=4,color='red')+

annotate('text',x=3.8,y=7,label='Aa',family='serif',

size=4,color='red')+

annotate('text',x=4.2,y=11.2,label='Ba',family='serif',

size=4,color='red')

ggarrange(p11,p22,p33,p44,p55,p66,ncol = 2,nrow = 3,common.legend = TRUE,legend = 'right',

labels = c('A','B','C','D','E','F'),

font.label = list(size=10,family='serif',color='black'))

**Nutrient restriction map visualization**

library(ggplot2)

df<-read.csv('N-P.csv',header = T)

#对于除了横纵轴之外的连续变量，可以映射到散点图的色深和点大小上

ggplot(df,aes(x=P,y=N,colour=type))+

geom_point()

#size绑定连续变量

ggplot(df,aes(x=P,y=N,colour=type,size=richness))+

geom_point()

#处理散点图重叠问题,"alpha"设置透明度

ggplot(df,aes(x=P,y=N,colour=type,size=richness))+

geom_point(alpha=.5)

#向散点图添加标签

library(gcookbook)

ggplot(df,aes(x=P,y=N,colour=type,size=richness))+

geom_point(alpha=.5)+

geom_text(aes(y=N+0.2,label=WZ))+

scale_x_continuous(limits = c(0,12.5))+

scale_y_continuous(limits =c(0,50))+

geom_abline(data=df_abline,aes(intercept=intercept,slope=slope,linetype=linetype))

#文本函数“geom_text(aes(y=N+0.2,label=WZ))”，y是指将原有坐标值向上偏移，其目的是让文本展示在样本点的上方而不是中间，label设置绑定文本

df_abline<-data.frame(intercept=c(0,0,0,0),slope=c(10,20,14,16),linetype=factor(c(1,1,1,1)))

#设置直线斜率属性

ggplot(df,aes(x=P,y=N,colour=type,size=richness))+

geom_point(alpha=.5)+

geom_text(aes(y=N+0.2,label=WZ))+

geom_abline(data=df_abline,aes(intercept=intercept,slope=slope,linetype=linetype))

Integrate the two graphs in AI software.

**correlation analysis**

library(corrplot)

par(mfrow=c(2,3))

d2<-read.csv('element2.csv',header = TRUE)#typeA

d1<-read.csv('element.csv',header = TRUE)#总的

d3<-read.csv('typeB.csv',header = TRUE)

d4<-read.csv('typeC.csv',header = TRUE)

e1<-cor(d1,method = 'spearman')

e2<-cor(d2,method = 'spearman')

e3<-cor(d3,method = 'spearman')

e4<-cor(d4,method = 'spearman')

g1<-cor.mtest(d1,conf.level=0.95)

g2<-cor.mtest(d2,conf.level=0.95)

g3<-cor.mtest(d3,conf.level=0.95)

g4<-cor.mtest(d4,conf.level=0.95)

corrplot(e1,p.mat = g1$p,type="upper",insig = 'label_sig',sig.level = c(.001,0.01,0.05),pch.cex = 0.9,

tl.cex = 0.5,order = 'AOE')

corrplot(e2,p.mat = g2$p,type="upper",insig = 'label_sig',sig.level = c(.001,0.01,0.05),pch.cex = 0.9,

tl.cex = 0.5,order = 'AOE')

corrplot(e3,p.mat = g3$p,type="upper",insig = 'label_sig',sig.level = c(.001,0.01,0.05),pch.cex = 0.9,

tl.cex = 0.5,order = 'AOE')

corrplot(e4,p.mat = g4$p,type="upper",insig = 'label_sig',sig.level = c(.001,0.01,0.05),pch.cex = 0.9,

tl.cex =0.5,order = 'AOE')

corrplot(e1,p.mat = g1$p,type="upper",insig = 'label_sig',sig.level = c(.001,0.01,0.05),pch.cex = 0.9,

tl.cex = 0.5)

corrplot(e2,p.mat = g2$p,type="upper",insig = 'label_sig',sig.level = c(.001,0.01,0.05),pch.cex = 0.9,

tl.cex = 0.5)

corrplot(e3,p.mat = g3$p,type="upper",insig = 'label_sig',sig.level = c(.001,0.01,0.05),pch.cex = 0.9,

tl.cex = 0.5)

corrplot(e4,p.mat = g4$p,type="upper",insig = 'label_sig',sig.level = c(.001,0.01,0.05),pch.cex = 0.9,

tl.cex =0.5)

**Principal component analysis and relative importance calculation**

library(vegan)

library(ggplot2)

b<-read.csv("typeC.csv")

a<-read.csv('pca222.csv')

#install.packages("factoextra")

library(factoextra)

pca<-prcomp(b,scale. = T)

y<-pca$x

pca$rotation

write.csv(y,file = 'pca222.csv')

summary(pca)

fviz_eig(pca)

fviz_pca_ind(pca)

fviz_pca_var(pca)+

theme_bw()+

theme_classic()+

xlab("Trait PC1 (45.9%)")+

ylab("Trait PC2 (37.13%)")

fviz_pca_biplot(pca)

relweights <- function(fit,...){

R <- cor(fit$model)

nvar <- ncol(R)

rxx <- R[2:nvar,2:nvar]

rxy <- R[2:nvar,1]

svd <- eigen(rxx)

evec <- svd$vectors

ev <- svd$values

delta <- diag(sqrt(ev))

lambda <- evec %*% delta %*% t(evec)

lambdasq <- lambda^2

beta <- solve(lambda) %*% rxy

rsquare <- colSums(beta^2)

rawwgt <- lambdasq %*% beta^2

import <- (rawwgt / rsquare)*100

lbls<-names(fit$model[2:nvar])

rownames(import)<-lbls

colnames(import)<-"Weights"

barplot(t(import),names.arg=lbls,

ylab="% of R-Square",

xlab="Predictor Variables",

main="Relative Importance of Predictor Variables",

sub=paste("R-Square=",round(rsquare,digits = 3)),

...)

return(import)

}

relweights(fit,col="lightgrey")

relweights(fit2,col="lightgrey")

write.csv(final_df,file = 'pca.csv')

Further visualization of images in Excel and AI software.
